# Periodontal Regeneration Using a Mineralizing Hydrogel Loaded with a Synthetic CEMP1-Derived Peptide

**DOI:** 10.3390/biom16101444

**Published:** 2026-10-03

**Authors:** Yu Wang, Guangzhao Guan, Li Mei, Huiwen Zheng, Xianghui Xing, Weibin Sun

**Affiliations:** 1 Department of Pediatric Dentistry, Nanjing Stomatological Hospital, Affiliated Hospital of Medical School, Institute of Stomatology, Nanjing University, Nanjing 210008, China; 602023350107@smail.nju.edu.cn (Y.W.);; 2 Department of Oral Diagnostic and Surgical Sciences, Faculty of Dentistry, University of Otago, Dunedin 9016, New Zealand; 3 Department of Oral Sciences, Faculty of Dentistry, University of Otago, Dunedin 9016, New Zealand; 4 Department of Periodontology, Nanjing Stomatological Hospital, Affiliated Hospital of Medical School, Institute of Stomatology, Nanjing University, Nanjing 210008, China

**Keywords:** periodontal regeneration, bioactive peptide, amorphous calcium phosphate (ACP), hydrogel biomaterials, osteogenic/cementogenic differentiation, TGF-β/Smad signaling

## Abstract

**Background**: Periodontal regeneration requires the coordinated reconstruction of mineralized and non-mineralized tissues. P35 polypeptide derived from Cementum protein-1(CEMP1) has been demonstrated to induce bio-mineralization in vitro, although in vivo applications still need to be further validated. In this study, we developed a novel mineralizing hydrogel system (P35/ACP@PEG Hy) to facilitate periodontal regeneration by encapsulating P35-loaded amorphous calcium phosphate nanoparticles (P35/ACP-NPs) within a four-arm polyethylene glycol (PEG) hydrogel. **Methods**: In vitro assays were performed to characterize the physicochemical properties, sustained-release behavior, osteogenic/cementogenic effects of P35/ACP-NPs on hPDLCs, together with the mechanical performance and biocompatibility of the PEG hydrogel. The regenerative capacity of the hydrogel was further assessed in a rat periodontal defect model using micro-CT and histological staining. The regenerative efficacy was evaluated in a rat periodontal defect model via micro-CT and histological staining, while transcriptome sequencing (RNA-seq) and immune-blotting were further performed to confirm the molecular mechanisms. **Results**: P35/ACP@PEG exhibited favorable physicochemical properties, featuring an initial burst release followed by a sustained gradual release profile, along with the excellent biocompatibility. In vitro, P35/ACP-NPs markedly promoted osteogenic and cementogenic differentiation of hPDLCs. Correspondingly, the P35/ACP@PEG hydrogel significantly accelerated periodontal tissue regeneration in the rat model with periodontal defect. And RNA-seq analysis and immune-blotting further revealed that these effects are highly linked to activation of the TGF-β/Smad2/3/LOX signaling pathway. **Conclusions**: P35/ACP@PEG Hy platform can promote periodontal regeneration via activation of TGF-β/Smad2/3/LOX signaling pathway in vivo. Its sustained bioactivity, favorable biocompatibility and robust regenerative efficacy highlight a potential of P35 polypeptide for periodontal tissue repair.

## 1. Introduction

Periodontitis is a common inflammatory disease characterized by progressive destruction of periodontal tissues, ultimately resulting in tooth mobility and loss [1,2]. While notable progress has been achieved in periodontal regenerative approaches, the complete restoration of periodontal function still represents a considerable clinical hurdle [3,4,5]. The challenge reflects the complex architecture of the periodontium, where cementum, alveolar bone and periodontal ligament (PDL) exhibit distinct spatial and temporal patterns of regeneration [6,7]. Bone, dentin, and cementum are mineralized tissues whose extracellular matrix (ECM) relies on type I collagen as its major structural scaffold. Approximately 50–70% of each tissue consists of carbonate substituted apatite. Their formation, osteogenesis, dentinogenesis and cementogenesis, begins with deposition of ECM, followed by a tightly regulated and sequential mineralization process [8].

Within the periodontium, this coordinated mineralization process depends on the structural and biochemical organization of the ECM. Collagen fibers form the interconnected scaffolding that supports the anatomical integration of cementum, PDL and alveolar bone. Their organized deposition, alignment and mineralization are essential for periodontal regeneration [9,10]. However, collagen fibers alone cannot initiate mineralization. Mineralization is regulated by non-collagenous proteins (NCPs), which bind to specific sites on collagen fibrils and control the nucleation and growth of calcium phosphate minerals. This interaction promotes both intrafibrillar and extrafibrillar mineralization [11,12]. NCPs therefore serve as a critical bridge mediating collagen-mineral interactions and play a pivotal role in biomimetic periodontal mineralization.

Cementum protein 1 (CEMP1) is predominantly expressed by cementoblasts and specific PDL cell subpopulations and functions as a key NCP regulating cementum mineralization [13,14]. CEMP1 promotes hydroxyapatite nucleation and growth in vitro and can drive cells from non-mineralizing to mineralizing states [15,16]. However, the extraction and purification of CEMP1 are technically challenging and costly, prompting the development of synthetic peptides that mimic its mineralization-regulating function. In our previous study, we designed a short peptide, P35, derived from CEMP1 based on a structure-guided molecular docking, in vitro mineralization assays and cell-based studies. P35 promoted intrafibrillar mineralization and enhanced osteogenic and cementogenic differentiation of hPDLCs [17]. Despite these promising findings, direct in vivo evidence supporting P35-mediated periodontal regeneration remains limited. Moreover, the short biological half-life and rapid clearance of peptides in vivo may restrict their therapeutic efficacy, highlighting the need for an effective delivery system capable of providing sustained and controlled P35 release within periodontal defects.

Calcium phosphate biomaterials are widely used in bone tissue engineering due to their biocompatibility, pH-responsive degradability and osteoinductive properties [18,19]. Among these materials, amorphous calcium phosphate (ACP) [Ca_3_(PO_4_)_2_·3H_2_O] offers several advantages over crystalline calcium phosphate-based materials, such as tricalcium phosphate (TCP) and hydroxyapatite (HA). ACP readily dissolves in physiological environments and can subsequently transform into apatite, providing locally available calcium and phosphate ions that support mineral deposition and osteogenic differentiation [20,21]. These properties also make ACP a promising carrier for bioactive molecules, enabling the integration of controlled therapeutic delivery with biomimetic mineralization. Such an approach is particularly relevant to periodontal regeneration, where conventional treatments can control inflammation and disease progression but remain limited in their ability to reconstruct the complex architecture of lost periodontal tissues [22]. Consequently, tissue engineering strategies that combine bioactive molecules with mineralizing scaffolds have attracted increasing attention for functional regeneration of periodontal defects [23,24,25].

Hydrogels have attracted considerable attention in tissue engineering due to their tunable hydrophilicity, biocompatibility and structural similarity to the natural ECM, enabling them to mimic physiological microenvironments and support cell growth or drug delivery [26,27]. Among hydrogel materials, tetra-armed polyethylene glycol (PEG) is particularly attractive because its terminal functional groups enable efficient crosslinking and precise network formation. By introducing functional groups at the terminals, PEG hydrogels can achieve enhanced crosslinking, superior mechanical strength and a more compact network structure suitable for tissue engineering applications [28,29].

**Figure 1:**
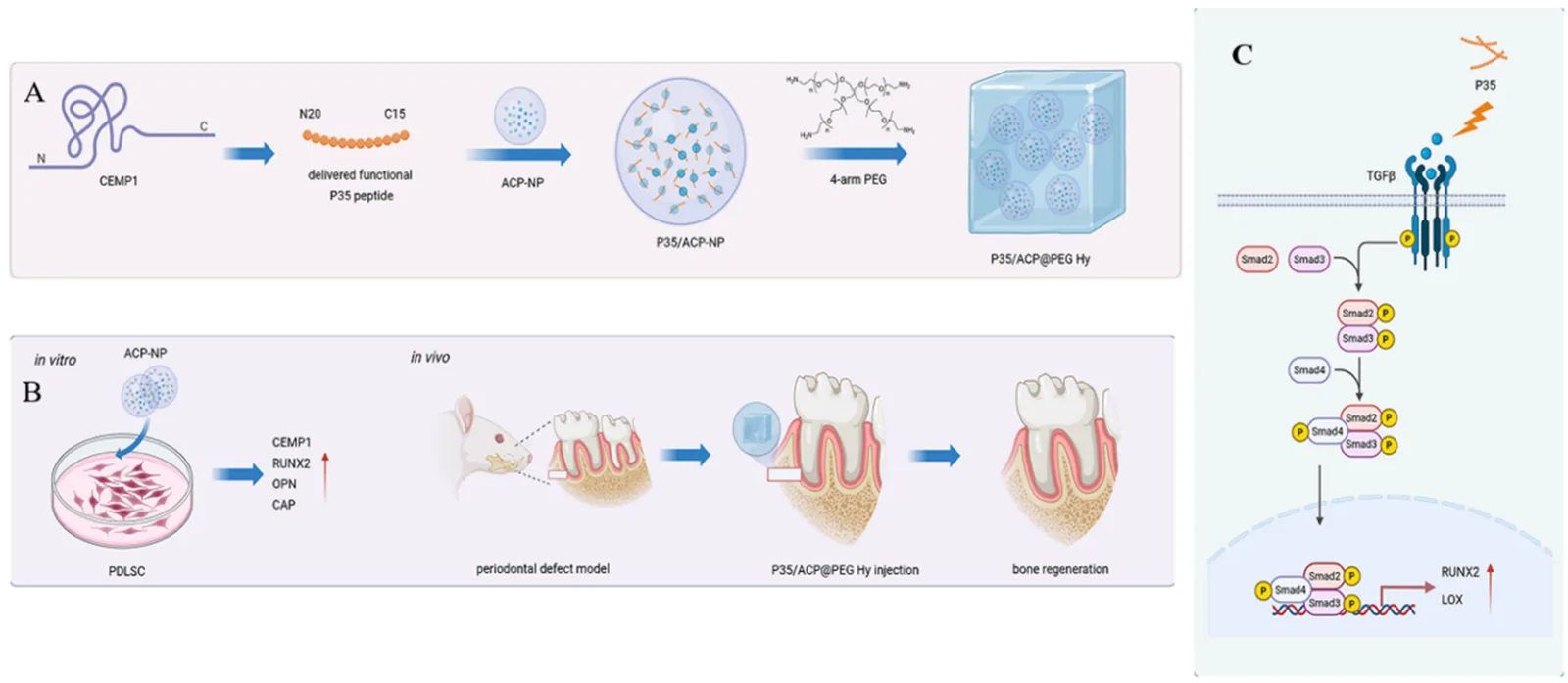
Scheme Design and functional mechanisms of P35/ACP@PEG Hy on the regulation of periodontal regeneration. (**A**) Schematic diagram of the design and synthesis of P35/ACP@PEG Hy. (**B**) In vitro: Regulatory effect of ACP-NP on the differentiation of hPDLCs. In vivo: Reparative effects of P35/ACP@PEG Hy on rat mandibular defects. (**C**) P35/ACP@PEG Hy modulates periodontal regeneration via the TGF-β/Smad2/3/LOX signaling pathway. Arrow indicated the upregulation of relative proteins.

Herein, we developed a hierarchically structured hydrogel by encapsulating P35-loaded amorphous calcium phosphate (ACP) nanoparticles within a four-arm polyethylene glycol (PEG) matrix. This system was designed to enable sustained peptide delivery while facilitating functional periodontal tissue regeneration. We systematically characterized its physicochemical properties, peptide release kinetics and cytocompatibility, while its effect on the osteogenic and cementogenic differentiation of hPDLCs was also investigated. The therapeutic efficacy of the hydrogel was further evaluated in a rat periodontal defect model. Together, these results establish a foundation for the development of P35-based biomimetic scaffolds for periodontal regeneration. A schematic illustration of the design and functional mechanisms of P35/ACP@PEG Hy for periodontal regeneration is shown in Figure 1.

## 2. Methods

Written informed consent was obtained from all the patients and the study was approved by the Ethics Committee of the Affiliated Stomatology Hospital of Nanjing University (Approval No. NJSH-2024NL-014).

### 2.1. Synthesis of P35 and PM

We synthesized P35 using standard solid-phase peptide synthesis, as described in our previous study [17]. Two short peptides derived from N-terminal 20 amino acids (sequences is MGTSSTDSQQAGHRRCSTSN) and C-terminal 15 amino acids (sequences is QGQGDTEDGRMTLMG) of CEMP1 were synthesized using the same protocol. Two short peptides were mixed at a mass ratio of 1:1 to generate a novel peptide termed as PM.

### 2.2. Preparation and Characterization of ACP Nanoparticles (ACP-NP)

ACP nanoparticles were prepared as described in a previous study [30]. Briefly, 2.22 g of PEG and 2.22 g of CaCl_2_ were dissolved in 200 mL of deionized water to achieve a 0.1 M solution of CaCl_2_. Then 5.056 g of Na_3_PO_4_·12H_2_O was dissolved in 100 mL of deionized water and stirred magnetically overnight, afterward the two solutions were mixed, followed by continuous magnetic stirring for 30 min, filtration and washing to remove chloride ions. Subsequently, P35 and PM solution was added to the calcium-phosphate solution, followed by magnetic stirring until complete dissolution, then the mixture was frozen for 2 days and stored at −18 °C under sealed conditions. The nanoparticles used in the study were termed as ACP-NP, P35/ACP-NP and PM/ACP-NP respectively.

### 2.3. Transmission Electron Microscopy (TEM) Observation

TEM (JEM-2100Plus, JEOL Ltd., Akishima, Tokyo, Japan) was used to observe the morphology of ACP nanoparticles with or without peptides.

### 2.4. X-Ray Diffraction (XRD)

Approximately 20 mg of different nanoparticles were dissolved in phosphate-buffered saline (PBS) at 37 °C, after thorough washing and drying, the samples were subjected XRD analysis to determine the diffraction peaks in the range of 20° < 2θ < 60° at the scanning speed of 2° per minute.

### 2.5. Fourier Transform Infrared (FTIR) Examination

The chemical structure of the nanoparticles was characterized by FTIR (IR Prestige-21, Shimadzu, Kyoto, Japan) with the scanning range of 4000–400 cm^−1^.

### 2.6. Controlled Release Assay of P35 from P35/ACP-NP

P35 aqueous solutions (20–600 μg/mL) were prepared for quantitative high-performance liquid chromatography (HPLC) analysis. A concentration standard curve was constructed by plotting P35 concentration on the X-axis versus peak area on the Y-axis. HPLC parameters were as follows: flow rate 1.0 mL/min, UV detection at 220 nm, injection volume 10 μL, and run time 25 min.

The peptide encapsulation efficiency (EE) and loading capacity (LC) were measured according to a previous study [31], in brief, the configured P35/ACP solution was placed in an ultrafiltration centrifuge tube and centrifuged at 5000 rpm at 4 °C for 20 min. Then the filtrate outside of the tube was collected, rinsed with PBS and the above steps were repeated. The concentration of P35 in the filtrate was determined by HPLC to calculate the mass of free P35. The encapsulation efficiency (EE) of P35 in the nanoparticles was calculated using the following formulas.

EE (%) = (*C_t_* − *C_f_*)/*C_t_* × 100%

where *C_t_* is the total weight of P35, *C_f_* is the weight of free P35 in the supernatant.

About 5 mL of P35/ACP solution subjected to ultrafiltration and centrifugation was immersed into 50 mL of PBS (pH 7.4) with a dialysis bag. At prespecified timepoints (1, 3, 7, 14, 21, and 28 days), an aliquot (1 mL) was removed and fresh medium of the same volume was added. The specimens were stored at −20 °C and the amount of P35 released into the medium was measured using HPLC. Three parallel samples were prepared for each group and the average value was taken.

### 2.7. Cytocompatibility Assay

The cytocompatibility of ACP-NP, PM/ACP-NP and P35/ACP-NP was evaluated using a CCK-8 assay. Briefly, hPDLCs were seeded in 96-well plates at 5000 cells per well and incubated for 24 h. The medium was then replaced with fresh medium (control) or containing nanoparticles at concentrations of 10, 50, and 100 μg/mL. After incubation for 1, 3, and 7 days, 10 μL of CCK-8 reagent was added to each well, followed by incubation at 37 °C for 2 h. The absorbance was measured at 450 nm using a microplate reader (Molecular Devices, Sunnyvale, CA, USA). All experiments were performed in triplicate. Data are presented as mean ± SD.

### 2.8. Isolation and Culture of Human hPDLCs

Teeth free caries or periodontal disease were collected from patients aged 16–20 years patients aged undergoing orthodontic treatment. The periodontal ligament tissue from the middle third of tooth roots was cut into 1 × 1 mm^2^ fragments and digested with 3 mg/mL Type I collagenase at 37 °C for 1 h. Cells were cultured in α-MEM (α-minimum essential medium) supplemented with 10% fetal ovine serum (FBS) and 1% penicillin-streptomycin (37 °C, 5% CO_2_) with medium refreshed every 2–3 days. Once the cells reached 80% confluence, passage 3–5 cells were used for subsequent experiments and maintained in DMEM (Dulbecco’s modified Eagle’s medium) under standard culture conditions.

### 2.9. Alizarin Red Staining (ARS)

After 14 days of culture in osteogenic medium (α-MEM, Gibco, Grand Island, NY, USA) supplemented with 0.1 μM dexamethasone (Gibco, USA), 50 μg/mL ascorbic acid (Gibco, USA) and 10 mM β-glycerophosphate (Gibco, USA) with or without different ACP nanoparticles, mineralized nodule formation by hPDLCs was assessed using 2% ARS. Cells were rinsed with deionized water, fixed with 70% ethanol for 1 h. Calcium deposits were then visualized using 2% ARS solution (Sigma–Aldrich, St. Louis, MO, USA). For quantitative analysis, the bound stain was extracted using CPC and mineralization was quantified according to the protocol described in our previous study.

### 2.10. Alkaline Phosphatase Activity (ALP) Assessment

After 14 days of culture in osteogenic induction medium with different ACP nanoparticle formulations, ALP in hPDLCs was assessed using a BCIP/NBT Alkaline Phosphatase Staining Kit (Beyotime Institute of Biotechnology, Shanghai, China) according to the manufacturer’s instructions. In brief, after discarding the culture medium, cells were rinsed by PBS and lysed using IP lysis buffer, then the supernatant was collected after centrifugation. ALP staining was performed according to the manufacturer’s instructions. Cells were incubated with the staining solution for 30 min at 37 °C, followed by visualization and image acquisition using a light microscopic. Relative ALP was quantified by measuring the absorbance at 562 nm. Experiments were performed in triplicate and repeated three times independently.

### 2.11. Real-Time Reverse Transcription-Polymerase Chain Reaction (RT-PCR)

hPDLCs were cultured in osteogenic differentiation medium with or without ACP nanoparticles for 7 or 14 days, total RNA of the cells was isolated via TRIzol^®^ Reagent (Ambion, Life Technologies, Carlsbad, CA, USA), cDNA was synthesized with the Hiscript III 1st Strand cDNA Synthesis Kit (R312-01, Vazyme, Nanjing, China). The gene expression associated with osteogenesis and cementogenesis was determined by iQTM SYBR Green Supermix (Q511-02, Vazyme, Nanjing, China). The expression levels of different mRNA were quantified by 2^−ΔΔCt^ method. The primer sequence for RT–qPCR was shown in Table S1.

### 2.12. Western Blot (WB) Analysis

In addition, after 7 or 14 days of culture in 24-well culture plates with or without ACP nanoparticles, Western blotting was performed to assess the protein expression of osteogenic and cementogenic markers, including RUNX2, OPN, CEMP1, and CAP. Cells were collected and lysed with RIPA lysis buffer to extract the total intracellular proteins. Proteins were separated by 10% SDS-PAGE and transferred onto PVDF membranes (Millipore Co., Billerica, MA, USA). After blocking with 5% non-fat milk solution overnight, membranes were incubated with primary antibodies overnight at 4 °C, then the membranes were washed three times with TBST, and incubated with secondary antibodies for 1 h at room temperature. Chemiluminescent reagent and a Western blot imaging system were used for detection, with β-actin as the loading control.

### 2.13. Synthesis and Characterization of PEG Hydrogel

The PEG hydrogel was fabricated according to a previously described method [28]. In brief, 25 mg of 4-arm poly (ethylene glycol) acrylate (MW: 20 k), 113 mg of acrylamide and 2.8 mg of lrgacure^TM^ 2959 were added into a 1 mL microcentrifuge tube with 100 µL of P35 or PM solution. The resultant mixture was homogenized by ultrasonic dispersion and subsequently photopolymerization under UV light (365 nm) for 3 min to form hydrogels functionalized with the different peptides (ACP@PEG Hy, P35/ACP@PEG Hy and PM/ACP@PEG Hy, respectively). Then, the hydrogels were dialyzed in PBS for 24 h to eliminate unreacted monomers and promote the complete swelling of hydrogels. All PEG precursors and PBS solutions were filtered through 0.22 μm sterile filters before use. The photopolymerization process under UV light was carried out in a sterile laminar flow hood and the resulting hydrogels were maintained under aseptic conditions.

### 2.14. Characterization of PEG Hydrogel

#### 2.14.1. Scanning Electron Microscope (SEM)

To observe the interior morphology of hydrogels from different groups, SEM images were obtained using a Scanning Electron Microscope (Quata200, FEI, Hillsboro, OR, USA) at an acceleration voltage of 10 kV. The samples were lyophilized and Au sputter-coated before the measurement.

#### 2.14.2. Rheologica Performance

To characterize the gelation time of the hydrogels, rheological measurements were conducted at 25 °C using a Stress Tech rheometer (TA Instruments, New Castle, DE, USA) with a cone-plate geometry (25 mm in diameter and 143 μm in gap distance) and frequency sweep as well as strain sweep tests were performed to comprehensively assess the viscoelastic behavior.

#### 2.14.3. Compressive Strength Analysis

The hydrogels from different groups were cut into cylindrical shape (thickness 5 mm, diameter 10 mm) and then subjected to the compressive strength test by a universal testing machine (Bose ELF 3220, Bose Corporation, Eden Prairie, MN, USA) in compression mode at a constant speed of 5 mm/min and the temperature is 25 °C.

### 2.15. Biocompatibility of PEG Hydrogels

10 mg freeze-dried hydrogels (PEG Hy, ACP@PEG Hy, PM/ACP@PEG Hy, and P35/ACP@PEG Hy, respectively) were immersed in 10 mL PBS for 24 h to prepare hydrogel extracts. hPDLCs (5 × 10^4^ cells/well) were seeded in a 96-well plate and maintained in osteogenic medium for 24 h. Cells were then cultured in fresh medium (control) or in medium containing extracts from the different hydrogels for 1, 3, and 7 days. CCK-8 assay was used to analyze the proliferation of hPDLCs according to the manufacturer’s protocol. Briefly, 10 μL of CCK-8 solution was added to each well for 2 h and then the absorbance was quantified at 450 nm by a Microplate Reader (Molecular Devices, Sunnyvale, CA, USA). To further evaluate cell biocompatibility, Live/Dead cell staining was performed using a cell staining assay (Solarbio, Beijing, China) after 5 days of incubation in different conditioned media. Briefly, cells were washed twice with PBS and incubated with 200 μL of staining solution containing Calcein-AM (2 μM) and propidium iodide (PI, 4 μM) at room temperature for 30 min in the dark. Live cells were stained green by Calcein-AM, while dead cells were stained red by PI. Fluorescence images were captured using a fluorescence microscope to visualize cell viability.

### 2.16. Animal Experiments

#### 2.16.1. Preparation of Rat Periodontal Defect Model

Forty adult male Wistar rats (aged 7–8 weeks, weighing 200–250 g) were obtained from the Institute of Laboratory Animal Center at Nanjing Agricultural University. They were divided into five groups (n = 8 in rats and the mucoperiosteal): (1) control (no treatment); (2) PEG Hy; (3) ACP@PEG Hy; (4) P35/ACP@PEG Hy; and (5) PM/ACP@PEG Hy. All animal experiments were conducted under strict aseptic surgical conditions and the hydrogel implants were sterilized by UV irradiation (30 min) before implantation. Following intraperitoneal anesthesia with 10% chloral hydrate, periodontal defects were surgically created in rats according to a previously described method [32]. A skin incision was made through the inferior border of the mandible in rats, 4 mm in width, and 1 mm and the mucoperiosteal flap was elevated to expose the buccal alveolar bone between inferior border of the mandible and the first and second molars. Thereafter, a periodontal bone defect (5 mm in length, 4 mm in width, and 1 mm in depth) was created on the bone surface covering the first and second molars using a high-speed dental turbine handpiece with cooling water. Then the periodontal ligament and cementum were fully removed. Based on the experimental protocol, the pre-cured hydrogel material was implanted into the defect region. The flap was then repositioned to achieve close adaptation to the root surface and secured with sutures, providing mechanical retention to hold the implanted material firmly in place. Eight rats from each group were euthanized at 4 weeks postoperatively for subsequent analysis. The mandibles were harvested, rinsed briefly with PBS and fixed in 4% paraformaldehyde overnight at 4 °C pending further analysis. For each group, all 8 mandibles were first scanned by micro-CT and the same specimens were subsequently processed for histological analysis (H&E staining). The sample size was determined based on previous literature and preliminary experiments.

#### 2.16.2. Micro-CT Analysis

The regeneration of the harvested rat mandibular alveolar bone was quantitatively analyzed by the microCT scanner (V6.5-3 Scanco Medical, Bruettisellen, Switzerland). The parameters were set as follows: 45 kVp scanning voltage, 180 μA scanning current, 12 μm resolution and 250 ms exposure time. Three-dimensional (3D) reconstructions were performed using CTAn software (v1.17.7.2, Bruker, Kontich, Belgium). Subsequently, quantitative morphometric analyses were conducted to calculate bone volume fraction (BV/TV), trabecular thickness (Tb.Th) and trabecular separation (Tb.Sp) within the periodontal defect.

#### 2.16.3. Histological Analysis

After imaging examination, the tissues were subjected to decalcification for 4 weeks followed by graded alcohol gradient dehydration. Subsequently, they were embedded in paraffin and sectioned into 4 μm-thick slices using a microtome and stained by hematoxylin and eosin (H&E) staining and Masson’s trichrome staining kits (Solarbio Science & Technology Co., Ltd., Beijing, China).

### 2.17. Mechanism of P35/ACP@PEG Hy-Mediated Periodontal Regeneration

To elucidate the molecular mechanisms underlying P35/ACP@PEG Hy mediated periodontal regeneration, transcriptome sequencing was conducted on hPDLCs cultured with and without P35/ACP@PEG Hy. Differentially expressed genes and enriched signaling pathways were subsequently identified to characterize the transcriptional regulatory networks activated by the hydrogel.

Based on the transcriptome sequencing results, the TGF-β/Smad2/3/LOX axis was identified as the dominant pathway. To verify its activation, the expression of key proteins in this cascade was assessed by Western blot analysis. Furthermore, a TGF-β receptor kinase inhibitor (SB-431542) and a Smad3 inhibitor (SIS3) were applied to confirm the regulatory role of the axis.

### 2.18. Statistical Analysis

Data are presented as mean ± standard deviation (SD). Statistical analyses were performed using SPSS version 19.0 (IBM Corp., Armonk, NY, USA). Multiple group comparisons were conducted by one-way analysis of variance (ANOVA), followed by Tukey’s post hoc test for pairwise comparisons. *p* value < 0.05 was considered as statistically significant.

## 3. Results

### 3.1. Characterization of ACP NPs

**Figure 2:**
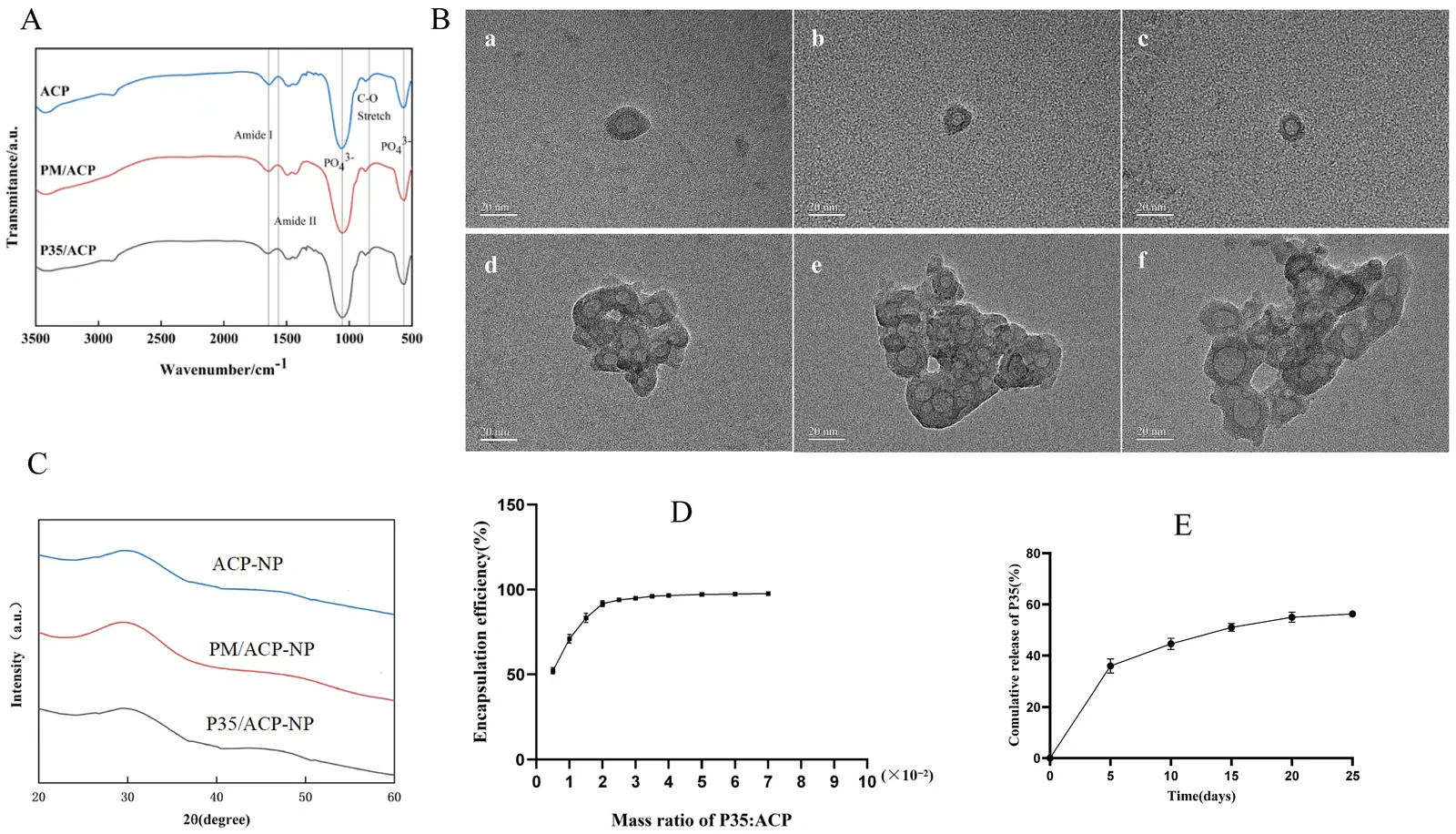
Characterization of different nanoparticles. (**A**) FTIR spectra. (**B**) TEM images of ACP-NP (**a**,**d**), PM/ACP-NP (**b**,**e**) and P35/ACP-NP (**c**,**f**), Scale bar: 20 nm. (**C**) XRD analysis. (**D**) Encapsulation efficiency of P35/ACP-NPs. (**E**) Cumulative release curve of P35 from P35/ACP-NPs.

Fourier transform infrared spectroscopy was conducted to analyze the chemical composition of the nanoparticles. The peaks at 590–610 cm^−1^ and ~1000 cm^−1^ are attributed to PO_4_^3−^ vibrations of amorphous calcium phosphate, while the broad absorption peak at 2886 cm^−1^ (CH_2_) and the stretching vibration peak at 842 cm^−1^ (C–O) are characteristic of PEG, confirming the successful synthesis of ACP nanoparticles. (Figure 2A). Moreover, the typical peaks at 1565 cm^−1^ and 1553 cm^−1^, which are attributed to the vibration of amide I and amide II vibrations, respectively, were observed in both PM/ACP-NP and P35/ACP-NP, confirming that PM or P35 has been successfully incorporated into the ACP nanoparticles. The nanoparticles in each group all exhibit a concentric circular structure with a diameter of 10–20 nm (Figure 2B(a–c)) and different degrees of agglomeration occur between the particles (Figure 2B(d–f)). The freeze-dried nanoparticles exhibit the characteristic peak of amorphous calcium phosphate at 30 degrees in XRD spectrum, all the results indicated the peptides have been encapsulated within ACP nanoparticles (Figure 2C). Based on the HPLC chromatograms of P35 at different concentrations, the standard calibration curve was constructed as illustrated in Figure 2D.

Within the concentration range of 20–600 μg/mL, a good linear correlation was observed between the peak area and P35 concentration. The linear regression equation was Y= 3.898X + 14.40, with a coefficient of determination (R^2^) of 0.999. Based on a signal-to-noise ratio (S/N) of 3:1 and 10:1, the limit of detection (LOD) and limit of quantification (LOQ) were determined to be 1.54 μg/mL and 5.13 μg/mL, respectively. All release samples were diluted appropriately prior to HPLC analysis, and their final concentrations were well above the LOQ. Recovery was assessed by spiking P35 into blank hydrogel extract at three concentration levels (20, 200 and 500 μg/mL), with three independent replicates prepared for each concentration. Samples were prepared and analyzed using the same procedure as described for the release study. Mean recoveries were 99.53% (RSD 1.24%), 98.48% (RSD 0.91%), 99.30% (RSD 0.66%) at 20, 200, and 500 μg/mL levels, respectively, confirming negligible peptide loss during the sample preparation. Inter-day repeatability was further evaluated over three consecutive days at the same three concentration levels (n = 3 per level per day), and the RSD values were 1.02%, 0.97%, and 0.75%, respectively, confirming excellent method reproducibility. These findings demonstrate that the established HPLC method is reliable and suitable for the accurate quantification of P35 in release studies.

The EE of P35/ACP-NP increased with increasing P35 loading, reaching a maximum EE of 97.4 ± 0.21% when the mass ratio of P35: ACP was 5–7 wt%. The in vitro release kinetics of P35 from P35/ACP-NP was illustrated in Figure 2E. A rapid release was observed in the first 5 days, with a cumulative release of 35.87 ± 2.80%. This was followed by a sustained and gradual release for up to 20 days with the cumulative release reaching 54.94 ± 1.96%. The in vitro cyto-compatibility of the nanoparticle formulations (ACP-NP, PM/ACP-NP, and P35/ACP-NP) was assessed by CCK-8 assay. As shown in Supplementary Figure S3, all three nanoparticle formulations (ACP-NP, PM/ACP-NP and P35/ACP-NP) showed no significant cytotoxicity over 7 days compared to the control, confirming their excellent biocompatibility.

### 3.2. In Vitro Osteogenic and Cementogenic Differentiation Assay

**Figure 3:**
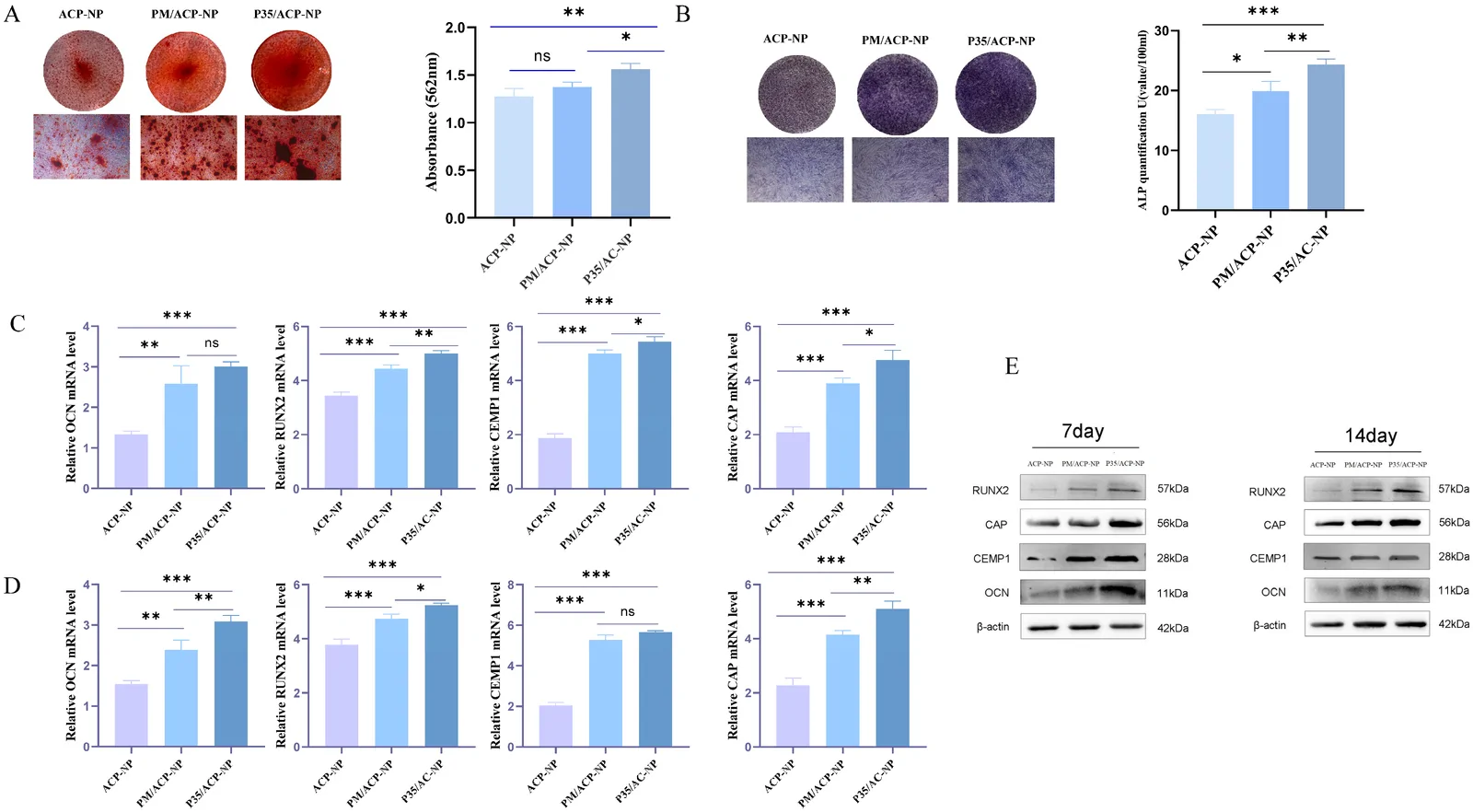
Osteogenic and cementogenic evaluation of different ACP-NPs. (**A**) Alizarin red staining and CPC quantitative analysis of mineralized nodules. (**B**) ALP staining and ALP detection. (**C**) and (**D**) RT-PCR analysis of OCN, RUNX2, CEMP1 and CAP expression after 7 days or 14 days of treatment, respectively. (**E**) Western blotting analysis of OCN, RUNX2, CEMP1 and CAP after incubation for 7 and 14 days. Mean values were compared by one-way ANOVA; * *p* < 0.05, ** *p* < 0.01, *** *p* < 0.001. Data were presented as mean ± SD (n = 3 independent biological replicates). Original Western blot images are provided in the Supplementary Materials.

The quantitative analysis was consistent with that of ALP, calcium deposition in the ACP-NP group was significantly lower than that in other groups, while P35/ACP-NP group showed the most prominent capacity to form mineralized nodules compared with other groups (*p* < 0.05) (Figure 3A). The ALP of hPDLCs cultured in different nanoparticle RUNX2, OCN, CEMP1, and CAP substrates after 7 days is shown in Figure 3B, among all the groups, P35/ACP-NP group exhibited the highest ALP, with a statistically significant difference compared with ACP-NP and PM/ACP-NP group (*p* < 0.05). We evaluated the osteogenic and cementogenic potential of different ACP-NP formulations by examining the mRNA and protein expression of RUNX2, OCN, CEMP1, and CAP in hPDLCs using RT-qPCR and Western blotting, respectively. The expression of the marker genes was significantly upregulated in the P35/ACP-NP group after 7 and 14 days of incubation compared with the ACP-NP and PM/ACP-NP groups (Figure 3C,D). In contrast, no statistically significant differences were observed between the PM/ACP-NP and ACP-NP group (*p* > 0.05). Consistent with the RT-qPCR results, Western blot analysis (Figure 3E) revealed that the P35/ACP-NP group exhibited the most pronounced upregulation of the corresponding proteins.

### 3.3. Microstructure, Mechanical Properties and Drug Release Behavior of PEG Hydrogels

**Figure 4:**
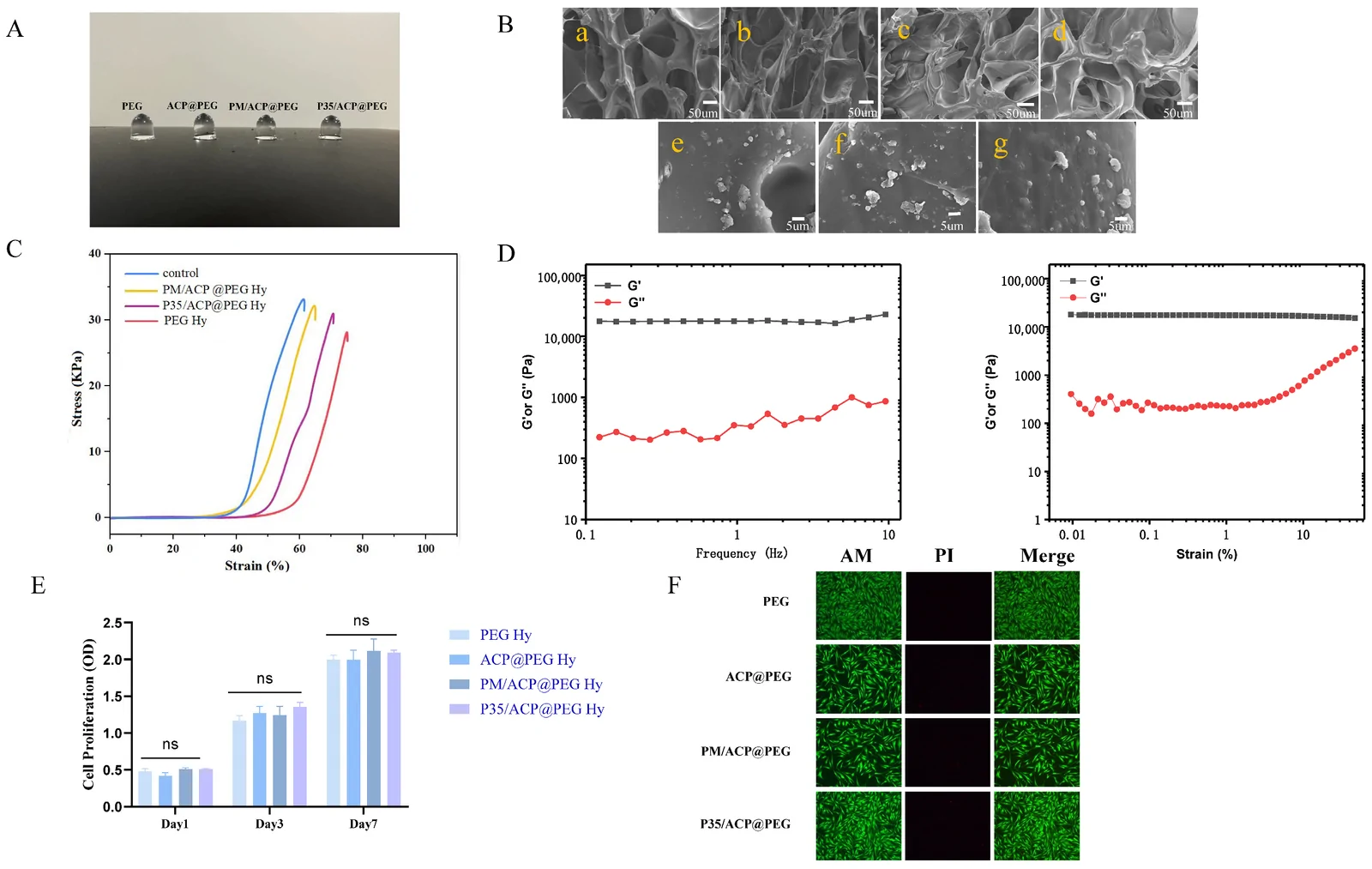
Characterization and biocompatibility of PEG Hydrogels (**A**) Macroscopic morphology of different hydrogels. (**B**) SEM images of the microstructure of the hydrogels ((**a**–**d**) belong to PEG Hy, ACP@PEG Hy, PM/ACP@PEG Hy and P35/ACP@PEG Hy, respectively; (**e**–**g**) are the magnified views of (**b**–**d**)) Scale bars: 50 μm (for (**a**–**d**)) and 5 μm (for (**e**–**g**)). (**C**) Stress–strain curve. (**D**) Rheological properties of the P35/ACP@PEG Hy. (**E**) hPDLCs cell viability measured using the CCK-8 assay. (**F**) Live/Dead cell staining of different hydrogels. Data are presented as mean ± SD, n = 3 per group.

All hydrogel formulations were homogeneous and transparent (Figure 4A). SEM showed that PEG hydrogels, with or without different ACP nanoparticles, exhibited a porous three-dimensional cross-linked network structure, with ACP nanoparticles embedded within the hydrogel matrix (Figure 4B). Specifically, the compressive strengths of the PEG, PM/ACP@PEG, P35/ACP@PEG, and ACP@PEG hydrogels were 32.42 ± 0.64, 28.05 ± 0.73, 31.28 ± 0.83, and 30.14 ± 0.92 kPa, respectively (Figure 4C). Rheological analysis of the P35/ACP@PEG hydrogel showed that the storage modulus (G′) remained higher than the loss modulus (G′′) throughout the frequency range of 0.1–10 Hz and the strain range of 0.01–15%, indicating excellent structural stability (Figure 4D). Moreover, the addition of ACP-NPs, irrespective of peptide functionalization, exerted little effect on the G′ and G′′ of the hydrogels (as shown in Table S2, Supplementary Data). These findings suggest that no complex chemical reactions occurred within the system, which is consistent with the compressive strength results. After 1, 2, and 3 days of incubation with different hydrogel extracts, the CCK-8 assay (Figure 4E) showed no significant differences among the test groups and the control group at any time point (*p* > 0.05). Furthermore, the live/dead staining images in Figure 4F suggested that the hydrogels exhibited no cytotoxicity to hPDLCs. Therefore, the results indicated that the hydrogels possessed favorable biocompatibility and no obvious cytotoxicity.

### 3.4. In Vivo Evaluation of Periodontal Repair and Regenerative Effects of PEG Hydrogel Formulations

**Figure 5:**
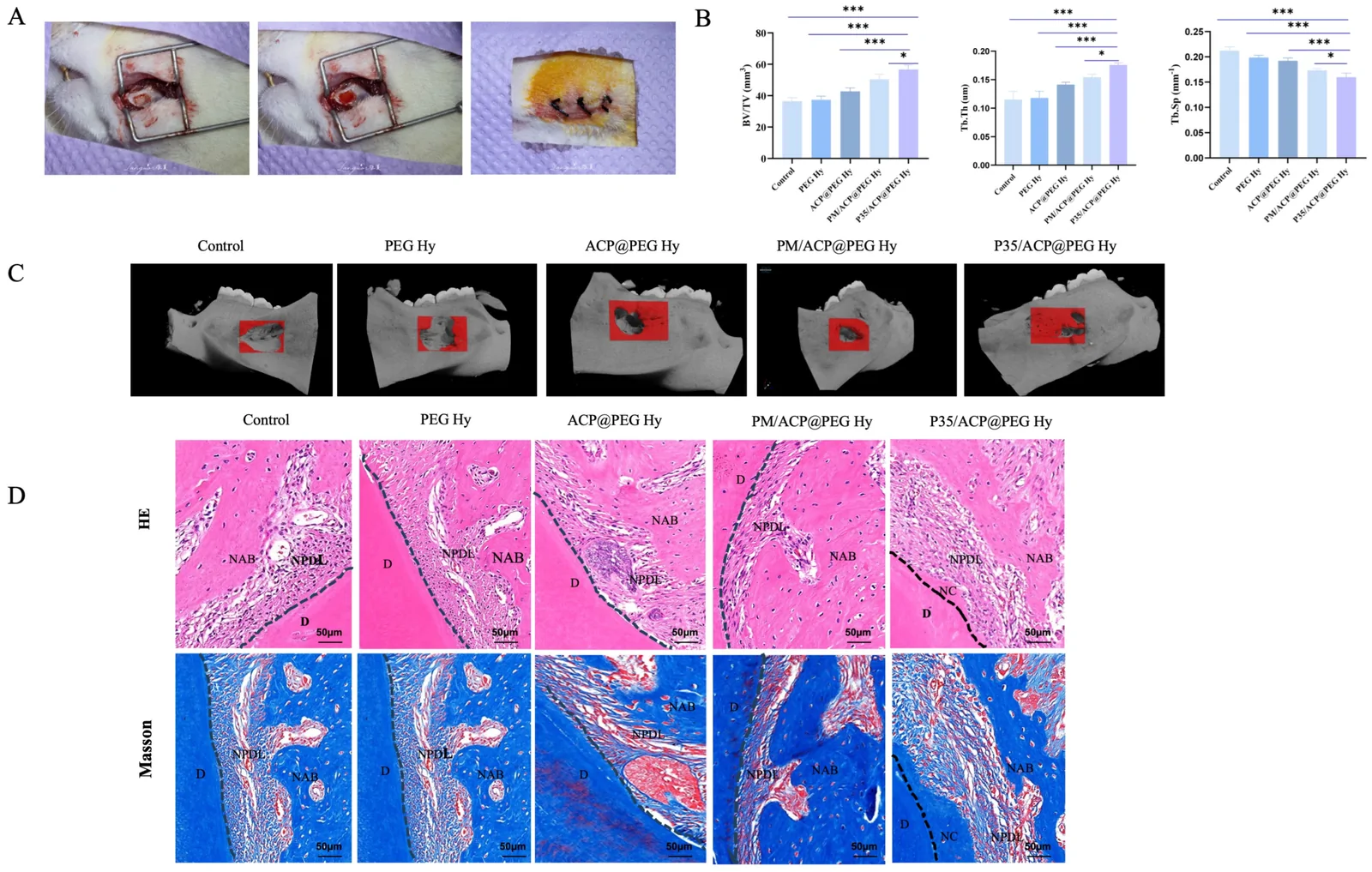
Periodontal repair and regenerative effects of different PEG Hydrogel Formulations after 4 weeks. (**A**) Scheme of surgical procedure. (**B**) Semi-quantitative analysis of bone-related parameters including BV/TV, Tb.Th and Tb.Sp. Data are presented as mean ± SD. n = 8 animals per group. Statistical significance was assessed by one-way ANOVA followed by Tukey’s post hoc test. (**C**) Micro-CT images of 3D reconstruction of periodontal tissues at 4 weeks (red region: defect area). (**D**) H&E and Masson’s trichrome staining of histological sections of periodontal defect at 4 weeks postoperatively (NAB, new alveolar bone; NPDL, new periodontal ligament; NC, new cementum; D, dentin). Scale bar = 50 µm (in (**D**)). * *p* < 0.05, and *** *p* < 0.001.

To investigate the effects of PEG hydrogel formulations on periodontal repair and regeneration, the periodontal defects of rats were treated with different interventions for 4 weeks. Micro-CT analysis (Figure 5C) revealed new bone formation in all groups. The control group presented the lowest quantity of newly formed bone, while the hydrogel-treated groups demonstrated significantly enhanced new bone formation (red areas) compared with that in the control group. Notably, the P35/ACP@PEG Hy group showed a remarkably higher amount of bone neoformation. Quantitative analysis presented in Figure 5B demonstrated the PM/ACP@PEG Hy and P35/ACP@PEG Hy groups presented significantly higher values of BV/TV and Tb.Th, accompanied by distinctly lower values of Tb.Sp in comparison with the remaining experimental groups. Furthermore, statistically significant differences were observed between the PM/ACP@PEG Hy and P35/ACP@PEG Hy groups (*p* < 0.05). Collectively, these findings indicate that the P35/ACP@PEG Hy group possessed a superior capacity to facilitate periodontal bone regeneration. HE and Masson staining revealed that all groups exhibited varying degrees of new bone formation at 4 weeks postoperatively (Figure 5D). Importantly, the periodontal ligament in the P35/ACP@PEG Hy group was denser and better-organized compared with control group. Moreover, regeneration of cementum was observed in the group treated with P35/ACP@PEG Hy.

### 3.5. Transcriptome Sequencing Analysis

**Figure 6:**
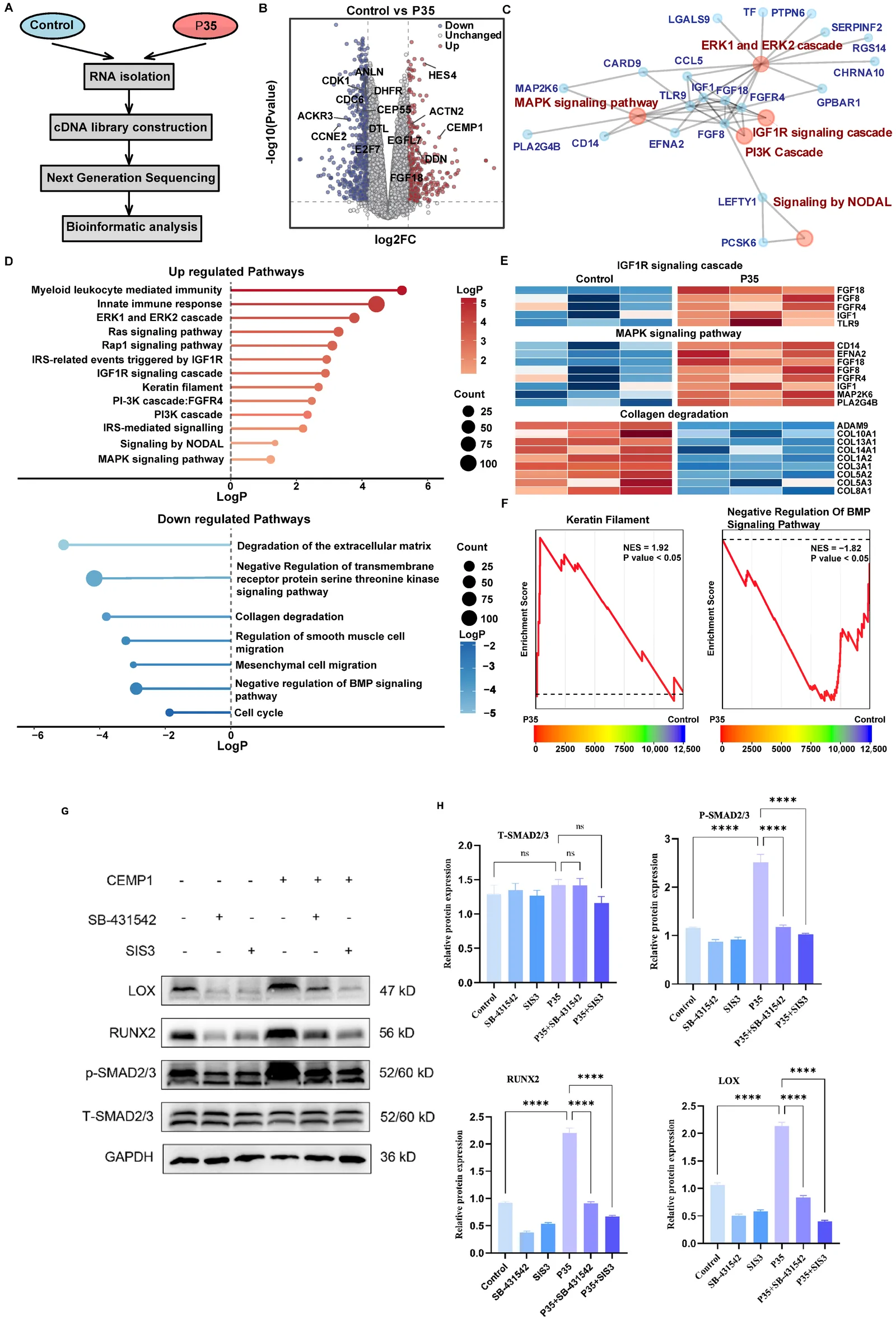
Sequencing analysis and validation of hPDLCs. (**A**) Sequencing process flowchart. (**B**) Volcano plot of differentially expressed genes (DEGs) in hPDLCs. The dashed vertical lines indicate the fold-change thresholds (|log2FC| = 1), and the dashed horizontal line represents the significance threshold (*p* = 0.05). (**C**) Protein–protein interaction (PPI) network analysis of hPDLCs. (**D**) KEGG pathway enrichment analysis of DEGs in hPDLCs. (**E**) Hierarchical clustering heatmap of DEGs in hPDLCs. (**F**) Gene set enrichment analysis of hPDLCs. (**G**) Western blot results indicating the expression levels of LOX, RUNX2 and phosphorylated Smad2/3. (**H**) Quantitative analysis of (**G**). Data are presented as mean ± SD (Data are presented as mean ± SD, n = 3 independent biological replicates). Statistical significance was assessed by one-way ANOVA followed by Tukey’s post hoc test **** *p* < 0.0001. Original Western blot images are provided in the Supplementary Materials.

To investigate the transcriptomic changes induced by P35 treatment, we performed RNA-seq analysis on control and P35-treated samples, followed by the bioinformatics interrogation (Figure 6A). Differential expression analysis identified a total of 1237 genes with significant expression changes (|log2FC| > 1, *p* < 0.05), where 628 genes were upregulated and 609 genes were downregulated (Figure 6B). Volcano plot visualization revealed prominent upregulation of genes such as HES4, ACTN2, DDN, CDK1 and CCNE2.

Protein–protein interaction (PPI) network analysis identified several important signaling pathways, including the ERK1, ERK2 and MAPK signaling pathway, IGF1R and PI3K signaling cascade and signaling by NODAL (Figure 6C). Consistent with these findings, pathway enrichment analysis revealed that upregulated genes were significantly associated with biological processes related to myeloid leukocyte-mediated immunity, innate immune response and the ERK1/ERK2 and IGF1R signaling cascades (Figure 6D).

Further heatmap analysis confirmed the differential regulation of core genes within these key pathways. Specifically, genes within the IGF1R and MAPK signaling cascades were predominantly upregulated, whereas genes involved in collagen degradation were down-regulated in P35-treated samples compared to controls (Figure 6E). Gene set enrichment analysis (GSEA) validated these findings, demonstrating significant enrichment of the Keratin Filament pathway (NES = 1.92, *p* < 0.05) in the control group. In contrast, the Negative Regulation of BMP Signaling Pathway (NES = −1.82, *p* < 0.05) was significantly enriched in the P35-treated group (Figure 6F).

To investigate the regulatory effects of CEMP1 on the TGF-β/Smad2/3 signaling pathway and osteogenic and cementogenic differentiation in hPDLCs, Western blot analysis was performed under various treatment conditions (as shown in Figure 6G). Compared with the control group, P35 treatment significantly increased the phosphorylation level of Smad2/3(p-Smad2/3), whereas total Smad2/3 expression remained unchanged. These results indicated that P35 specifically activated the canonical TGF-β/Smad2/3 signaling pathway. In parallel, P35 exposure markedly increased the expression of RUNX2 and LOX, implying that CEMP1 contributes to osteogenic and cementogenic differentiation of hPDLCs by engaging the TGF-β/Smad2/3 signaling cascade.

To further validate the dependency on this pathway, two specific pharmacological inhibitors, SB-431542 (a selective TGF-β type I receptor inhibitor) and SIS3 (a specific Smad3 inhibitor), were employed. Western blot revealed that compared with the control group, P35 treatment significantly increased the expression of p-Smad2/3, RUNX2, and LOX (all *p* < 0.0001). Co-treatment with either SB-431542 or SIS3 significantly suppressed P35-induced p-Smad2/3 expression (*p* = 0.9983 and *p* = 0.2793, respectively). Consistently, both inhibitors markedly reversed the enhanced expression of RUNX2 and LOX elicited by P35 (all *p* < 0.0001), confirming that the regulatory effects of P35 on osteogenic and cementogenic differentiation were mediated via the TGF-β/Smad2/3/LOX signaling axis.

## 4. Discussion

The present study investigated the potential of P35 as a bioactive agent for periodontal regeneration in vivo. P35 stability and local delivery were enhanced by encapsulating the peptide within ACP-NPs and incorporating them into a tetra-armed PEG hydrogel. This hierarchical system reduced burst release and degradation, enabling sustained P35 release while preserving the rheological and mechanical properties of the hydrogel. The functional importance of the intact P35 peptide was further evaluated by comparison with a physically mixed peptide (PM) containing its HAP-binding and collagen-interacting domains. Although PM/ACP-NPs promoted osteogenic and cementogenic differentiation of hPDLCs, their effects were weaker than those of P35/ACP-NPs. Consistently, in vivo bone regeneration was greatest with P35/ACP@PEG Hy, followed by PM/ACP@PEG Hy and ACP@PEG Hy, supporting the superior regenerative activity of intact P35.

In recent years, bioinspired peptides have attracted increasing attention in periodontal tissue engineering. Compared with natural proteins, synthetic peptides possess several advantages, including simplified synthesis procedures, lower production costs, improved biosafety and stable physicochemical properties. Moreover, peptides could be rationally designed or modified to achieve specific functional requirements. Their relatively small molecular size also facilitates tissue penetration and enables efficient loading onto various biomaterial carriers for localized delivery [33,34,35]. Despite these advantages, studies exploring CEMP1 derived biomimetic peptides in periodontal tissue engineering remain limited. Most reported peptides represent only partial fragments of CEMP1. Although these fragments demonstrate calcium–phosphate binding affinity and potential mineralization-promoting activity [36,37,38], the in vivo regenerative efficacy and the underlying molecular mechanisms remain largely unexplored.

The superior regenerative capacity of P35 compared with the physically mixed peptides could be attributed to fundamental differences in molecular architecture and functional coordination. In P35, the two functional domains are covalently linked within a single peptide backbone, enabling the formation of a stable spatial conformation that facilitates synergistic interactions between the collagen-binding and mineralization-regulating domains [39,40]. The structural integration allows P35 to efficiently mediate hydroxyapatite nucleation, collagen binding and the orderly regulation of mineral deposition. In contrast, the physically mixed peptides rely only on random intermolecular interactions and lack a defined spatial orientation, making it difficult to achieve coordinated multi-target binding and effective mineralization templating. Furthermore, the covalent tandem structure of P35 likely enhances peptide stability and local retention within the microenvironment, whereas the individual fragments in the mixture may diffuse or degrade more rapidly, resulting in reduced biological activity [41,42]. These findings suggest that the enhanced functionality of P35 arises not from simple sequence combination but from the synergistic effect generated through structural integration.

Transcriptomic analysis further revealed that P35 treatment induced substantial changes in gene expression profiles associated with tissue repair and ECM synthesis. Specifically, genes involved in ECM production and matrix organization were significantly upregulated, while pathways related to cell cycle progression and inflammatory signaling were suppressed. These transcriptional changes suggest that P35 promotes a phenotypic transition toward a reparative, matrix-secreting cellular state that is favorable for periodontal tissue regeneration.

The upregulation of HES4, a downstream effector of the Notch signaling pathway, together with activation of IGF1R- and MAPK- related pathways, suggests potential crosstalk between Notch signaling and the TGF-β superfamily signaling network. Such interactions are known to facilitate differentiation toward fibroblastic and osteoblastic lineages, which are essential for effective tissue repair [43]. Additionally, the increased expression of ACTN2 and DDN indicates enhanced cytoskeletal organization, which may support lysyl oxidase (LOX)-mediated ECM maturation and stabilization [44].

Conversely, the downregulation of key cell cycle regulators, including CDK1 and CCNE2, together with reduced expression of inflammatory chemokine receptors such as ACKR3, indicates a shift from a proliferative state toward a functional matrix-producing phenotype [45,46]. This transition is characteristic of TGF-β- mediated tissue repair process, where cells prioritize matrix synthesis and remodeling rather than rapid proliferation. The suppression of genes associated with ECM degradation further supports the establishment of a stable microenvironment conducive to tissue regeneration.

hPDLCs possess strong self-renewal capacity and multilineage differentiation potential and their osteogenic/cementogenic differentiation plays a crucial role in periodontal tissue regeneration [47]. In this study, we further investigated the molecular mechanisms underlying P35-induced osteogenic and cementogenic differentiation of hPDLCs. Pretreatment with specific pharmacological inhibitors markedly abolished the P35-induced upregulation of RUNX2 and LOX, confirming that the osteogenic/cementogenic effects of P35 are highly dependent on activation of the TGF-β/Smad2/3 signaling pathway. RUNX2 and LOX were identified as key downstream effector molecules in this process. However, it should be noted that while in vitro inhibitor experiments support the involvement of TGF-β/Smad signaling in P35-mediated regeneration, direct causal evidence in the in vivo defect model (e.g., through local administration of TGF-β pathway inhibitors) is still lacking. Future studies employing pharmacological inhibition or genetic approaches in vivo will be necessary to definitively establish the contribution of this axis in periodontal tissue regeneration.

The TGF-β/Smad2/3 pathway is a canonical signaling axis involved in regulating osteogenic/cementogenic differentiation in various stem cell populations, including hPDLCs [32]. Consistent with this established role, P35 treatment significantly increased Smad2/3 phosphorylation, thereby initiating the osteogenic and cementogenic differentiation program. RUNX2, widely regarded as a master transcriptional regulator of osteogenesis, controls the expression of numerous osteogenic and cementogenic genes [48]. Meanwhile, LOX plays a crucial role in ECM maturation by catalyzing crosslinking of collagen fibers, thereby enhancing matrix stability and mechanical strength [49]. Notably, while we identified the TGF-β/Smad/LOX axis as a key mechanism underlying P35/ACP@PEG hydrogel-mediated periodontal regeneration, RNA-seq profiling concurrently revealed enrichment of other well-established pathways, including ERK/MAPK, IGF1R, and BMP signaling, which have been extensively documented in periodontal tissue repair [50,51,52]. The precise crosstalk between the TGF-β/Smad/LOX axis and these pathways is currently under systematic investigation.

Based on these findings, we suggest that P35 exerts a dual regulatory function during periodontal regeneration. First, it activates RUNX2 to initiate the osteogenic and cementogenic differentiation program in hPDLCs. Second, it upregulates LOX expression to promote collagen crosslinking and ECM stabilization, thereby creating a supportive microenvironment for sustained tissue regeneration. This coordinated regulation of both cellular differentiation and matrix maturation represents a key mechanistic feature underlying the regenerative capacity of P35.

Of course, several limitations should be considered. First, cementum and periodontal ligament regeneration in this study were mainly based on histological observations, lacking systematic quantitative analysis and immunohistochemical validation with specific markers (e.g., CEMP1, CAP). Second, it should be acknowledged that periodontal regeneration is a spatiotemporally coordinated process involving sequential and interlinked biological events including inflammation resolution, angiogenesis and extracellular matrix remodeling that are regulated in a defined temporal manner [53]. The current single-factor P35 release from the P35/ACP@PEG hydrogel represents a simplified model that does not fully recapitulate this complexity and has shown limited clinical efficacy in complex pathological states. Consequently, multi-modal release strategies capable of delivering multiple bioactive factors in a spatially and temporally controlled fashion have been demonstrated as effective methodologies to modulate distinct stages of tissue healing [54]. Future work should explore sequential co-delivery systems (e.g., combining P35 with angiogenic or anti-inflammatory agents) to better mimic the native regenerative cascade and further enhance therapeutic outcomes. Third, the animal study included only a single 4-week endpoint, which may not fully capture late-stage bone maturation, cementum apposition and PDL remodeling; validation in large-animal models is also needed. Fourth, further optimization of the hydrogel scaffold, particularly regarding its mechanical properties to better match the stiffness gradient of native periodontal tissues may improve its adaptability to the dynamic process of periodontal tissue regeneration.

## 5. Conclusions

The hierarchical P35/ACP@PEG hydrogel developed in this study effectively facilitates functional periodontal regeneration, which may involve activation of the TGF-β/Smad2/3/LOX signaling pathway. With its excellent biocompatibility, sustained bioactivity and robust regenerative capacity, this biomimetic delivery system represents a promising therapeutic strategy for periodontal defect repair and regeneration. Finally, we acknowledge that the translation of therapeutic hydrogels is often challenged by the lack of transparency in preclinical data. To accelerate clinical progress, future studies should prioritize the sharing of both successful and unsuccessful cases, as negative data regarding in vivo failures are equally crucial for optimizing next-generation materials.

## Data Availability

All data supporting the findings of this study are available within the manuscript and its Supplementary Information Files. On reasonable request, the corresponding author will provide the data and code conclusions of this work.

## Supplementary Materials

The following supporting information can be downloaded at: https://www.mdpi.com/article/10.3390/biom16101444/s1. Figure S1: Frequency sweep test and Strain sweep test of PEG Hy, ACP@PEG Hy and PM@PEG Hy. Figure S2: High-performance liquid chromatography (HPLC) and mass spectrometry (MS) analysis of the short peptides derived from the N terminal 20 amino acids and C-terminal amino acids of CEMP1. Figure S3: Cytocompatibility of nanoparticle formulations assessed by CCK-8 assay. Figure S4: RT-PCR analysis of OCN, RUNX2, CEMP1 and CAP expression after 7 days of treatment with PEG Hy, ACP@PEG Hy, PM/ACP@PEG Hy, and P35/ACP@PEG Hy, respectively. Table S1: The primer sequence for RT–qPCR in PDLSCs treated with different nanoparticles. Table S2: Micro-CT parameters (mean ± SD, n = 8 per group) comparing P35/ACP@PEG Hy and PM/ACP@PEG Hy. Figure S5: The original Western Blot membrane images of osteogenesis and cementogenesis related protein expression after incubation for 7 days. Figure S6 The original Western Blot membrane images of osteogenesis and cementogenesis related protein expression after incubation for 14 days. Figure S7: The original Western Blot membrane images of Figure 6.

## References

[1] Lockhart P.B., Brennan M.T., Thornhill M., Michalowicz B.S., Noll J., Bahrani-Mougeot F.K., Sasser H.C. (2009). Poor oral hygiene as a risk factor for infective endocarditis-related bacteremia. J. Am. Dent. Assoc..

[2] Kinane D.F., Stathopoulou P.G., Papapanou P.N. (2017). Periodontal diseases. Nat. Rev. Dis. Primers.

[3] Liu J., Ruan J., Weir M.D., Ren K., Schneider A., Wang P., Oates T.W., Chang X., Xu H.H.K. (2019). Periodontal Bone-Ligament-Cementum Regeneration via Scaffolds and Stem Cells. Cells.

[4] Zhang Y., Ren J., Shen Z., Yang J., Yang J., Lin Z., Shi X., Zhao C., Xia J. (2025). BMP2 peptide-modified polycaprolactone-collagen nanosheets for periodontal tissue regeneration. Front. Bioeng. Biotechnol..

[5] Chen P., Zhang C., He P., Pan S., Zhong W., Wang Y., Xiao Q., Wang X., Yu W., He Z. (2022). A Biomimetic Smart Nanoplatform as "Inflammation Scavenger" for Regenerative Therapy of Periodontal Tissue. Int. J. Nanomed..

[6] Liu G., Xue J., Zhou X., Gui M., Xia R., Zhang Y., Cai Y., Li S., Shi S., Mao X. (2025). The paradigm shifts of periodontal regeneration strategy: From reparative manipulation to developmental engineering. Bioact. Mater..

[7] Ono Y., Kaku M., Thant L., Iwama H., Arai M., Mizukoshi M., Dobashi A., Kitami M., Taketo M.M., Ohazama A. (2025). Wnt/β-catenin Promotes Cementum Apposition in Periodontal Regeneration. J. Dent. Res..

[8] Salmon C.R., Giorgetti A.P.O., Paes Leme A.F., Domingues R.R., Kolli T.N., Foster B.L., Nociti F.H. (2017). Microproteome of dentoalveolar tissues. Bone.

[9] Hu L., Cheng D., Yuan X., Gao Z., Yi Q., Zhao B., Wei F., Xu J., Fan Z., Liu Y. (2024). Engineered pre-dentin with well-aligned hierarchical mineralized collagen fibril bundles promote bio-root regeneration. J. Tissue Eng..

[10] Yu M., Luo D., Qiao J., Guo J., He D., Jin S., Tang L., Wang Y., Shi X., Mao J. (2021). A hierarchical bilayer architecture for complex tissue regeneration. Bioact. Mater..

[11] Xu J., Chen Y., Li X., Lei Y., Shu C., Luo Q., Chen L., Li X. (2021). Reconstruction of a Demineralized Dentin Matrix via Rapid Deposition of CaF_2_ Nanoparticles *In Situ* Promotes Dentin Bonding. ACS Appl. Mater. Interfaces.

[12] Yu L., Wei M. (2021). Biomineralization of Collagen-Based Materials for Hard Tissue Repair. Int. J. Mol. Sci..

[13] Villarreal-Ramírez E., Moreno A., Mas-Oliva J., Chávez-PachecoJ L., Narayanan A.S., Gil-Chavarría I., Zeichner-David M., Arzate H. (2009). Characterization of recombinant human cementum protein 1 (hrCEMP1): Primary role in biomineralization. Biochem Biophys. Res. Commun..

[14] Wang Q.Q., Miao L., Zhang H., Wang S.Q., Li Q., Sun W. (2020). A novel amphiphilic oligopeptide induced the intrafibrillar mineralisation via interacting with collagen and minerals. J. Mater. Chem. B.

[15] Arroyo R., López S., Romo E., Montoya G., Hoz L., Pedraza C., Garfias Y., Arzate H. (2020). Carboxy-Terminal Cementum Protein 1-Derived Peptide 4 (cemp1-p4) Promotes Mineralization through wnt/β-catenin Signaling in Human Oral Mucosa Stem Cells. Int. J. Mol. Sci..

[16] Bermúdez M., Imaz-Rosshandler I., Rangel-Escareño C., Zeichner-David M., Arzate H., E Mercado-Celis G. (2015). CEMP1 Induces Transformation in Human Gingival Fibroblasts. PLoS ONE.

[17] Wang Y., Mei L., Zheng H., Xing X., Sun W. (2025). A novel mineralization-inductive peptide derived from CEMP1functinal domains. Sci. Rep..

[18] Niu X., Chen S., Tian F., Wang L., Feng Q., Fan Y. (2016). Hydrolytic conversion of amorphous calcium phosphate into apatite accompanied by sustained calcium and orthophosphate ions release. Mater. Sci. Eng. C.

[19] Tolmacheva N., Bhattacharyya A., Noh I. (2024). Calcium Phosphate Biomaterials for 3D Bioprinting in Bone Tissue Engineering. Biomimetics.

[20] Samavedi S., Whittington A.R., Goldstein A.S. (2013). Calcium phosphate ceramics in bone tissue engineering: A review of properties and their influence on cell behavior. Acta Biomater..

[21] Bohner M. (2000). Calcium orthophosphates in medicine: From ceramics to calcium phosphate cements. Injury.

[22] Yamada S., Shima N., Kitamura H., Sugito H. (2002). Effect of porous xenographic bone graft with collagen barrier membrane on periodontal regeneration. Int. J. Periodontics Restor. Dent..

[23] Zhang C., Liu C., Liaw A., Ivanovski S., Han P. (2025). Synergistic potentials of small extracellular vesicles, biomaterials, and 3D bioprinting in periodontal regeneration: A scoping review. Extracell. Vesicles Circ. Nucl. Acids.

[24] Qiu P., Ouyang Y., Liu S., Dai J., Wang R., Zhao W., Xu C., Fan Z. (2026). Environmental Response Temporal Release Injectable Hydrogel for Controlled Growth Factor Release to Enhance Inflammatory Periodontal Bone Defect Regeneration. Adv. Mater..

[25] Qiu W., Zhang K., Wu M., Fu M., Wu H., Tang R., Chen Z., Guo J., Fang F. (2025). Tri-Layer Citrate-Based Hydroxyapatite Composite Scaffold Promoting Osteogenesis and Gingival Tissue Regeneration for Periodontal Bone Defect Repair. Adv. Healthc. Mater..

[26] Fang G., Zhao R., Zhu L., Wang Q., Peng S., Kang L., Lu H., Zhang G., Tang B. (2025). Nanoemulsion-based pseudopolyrotaxane hydrogel for enhanced corneal bioavailability and treatment of corneal inflammation. J. Control Release.

[27] Xia Y., Chen Z., Zheng Z., Chen H., Chen Y. (2024). Nanomaterial-integrated injectable hydrogels for craniofacial bone reconstruction. J. Nanobiotechnol..

[28] Wei Y., Tang J., Li J., Hou X., Li L., Zhang D., Chai D., Zhao M., Liu F. (2023). A novel tetra-PEG based hydrogel for prevention of esophageal stricture after ESD in a porcine model. Colloids Surf. B Biointerfaces.

[29] Bu Y., Zhang L., Sun G., Sun F., Liu J., Yang F., Tang P., Wu D. (2019). Tetra-PEG Based Hydrogel Sealants for In Vivo Visceral Hemostasis. Adv. Mater..

[30] Chen X., Liu Y., Miao L., Wang Y., Ren S., Yang X., Hu Y., Chen X. (2016). Controlled release of recombinant human cementum protein 1 from electrospun multiphasic scaffold for cementum regeneration. Int. J. Nanomed..

[31] Hu D., Tian T., Ren Q., Han S., Li Z., Deng Y., Lu Z., Zhang L. (2024). Novel biomimetic peptide-loaded chitosan nanoparticles improve dentin bonding via promoting dentin remineralization and inhibiting endogenous matrix metalloproteinases. Dent. Mater..

[32] Cui S.-J., Fu Y., Yu M., Zhang L., Zhao W.-Y., Zhang T., Qiu L.-X., Gu Y., Zhou Y.-H., Liu Y. (2022). Functional periodontal regeneration using biomineralized extracellular matrix/stem cell microspheroids. Chem. Eng. J..

[33] Kao D.J., Hodges R.S. (2009). Advantages of a synthetic peptide immunogen over a protein immunogen in the development of an anti-pilus vaccine for Pseudomonas aeruginosa. Chem. Biol. Drug Des..

[34] Yu D., Han N., Son H., Kim S.J., Kweon S. (2026). Functional Peptide-Based Biomaterials for Pharmaceutical Application: Sequences, Mechanisms, and Optimization Strategies. J. Funct. Biomater..

[35] Diller D.J., Swanson J., Bayden A.S., Jarosinski M., Audie J. (2015). Rational, computer-enabled peptide drug design: Principles, methods, applications and future directions. Future Med. Chem..

[36] Campos M.N., Giraldo E.L., Del Rio Portilla F., Fernández-Velasco D.A., Arzate H., Romo-Arévalo E. (2023). Solution NMR structure of cementum protein 1 derived peptide (CEMP1-p1) and its role in the mineralization process. J. Pept. Sci..

[37] Hoz L., López S., Zeichner-David M., Arzate H. (2021). Regeneration of rat periodontium by cementum protein 1-derived peptide. J. Periodontal Res..

[38] Montoya G., Correa R., Arenas J., Hoz L., Romo E., Arroyo R., Zeichner-David M., Arzate H. (2019). Cementum protein 1-derived peptide (CEMP 1-p1) modulates hydroxyapatite crystal formation in vitro. J. Pept. Sci..

[39] Cloyd A.K., Boone K., Ye Q., Snead M.L., Spencer P., Tamerler C. (2023). Engineered Peptides Enable Biomimetic Route for Collagen Intrafibrillar Mineralization. Int. J. Mol. Sci..

[40] Fukuhara H., Sato Y.T., Hou J., Iwai M., Todo T. (2023). Fusion peptide is superior to co-expressing subunits for arming oncolytic herpes virus with interleukin 12. Commun. Med. (Lond.).

[41] Castro V.I.B., Araújo A.R., Reis R.L., Pashkuleva I., Pires R.A. (2025). Nanoengineered Self-Assembling Peptides with Increased Proteolytic Stability Promote Wound Healing. ACS Appl. Mater. Interfaces.

[42] Guo X., Ma Y., Ruhan A., Pan S., Guo Y., Shi X., Gao G., Sun L., Wang J. (2022). Functionalization of biomedical materials using fusion peptides for tissue regeneration. BioMed. Mater..

[43] Bakalenko N., Kuznetsova E., Malashicheva A. (2024). The Complex Interplay of TGF-β and Notch Signaling in the Pathogenesis of Fibrosis. Int. J. Mol. Sci..

[44] Curtis A.J., Zhu J., Penny C.J., Gold M.G. (2023). Molecular basis of interactions between CaMKII and α-actinin-2 that underlie dendritic spine enlargement. Elife.

[45] Takaya K., Asou T., Kishi K. (2022). Selective Elimination of Senescent Fibroblasts by Targeting the Cell Surface Protein ACKR3. Int. J. Mol. Sci..

[46] Gorjala P., Cairncross J.G., Gary R.K. (2016). P53-dependent up-regulation of CDKN1A and down-regulation of CCNE2 in response to beryllium. Cell Prolif..

[47] Zhao Z., Chen L., Zhu S., Yu H., Chen Y., Song J., Liu Y., He D. (2025). Periodontal ligament stem cells in tissue remodeling: From mechanical forces to inflammatory signals. Stem Cell Res. Ther..

[48] Komori T. (2019). Regulation of Proliferation, Differentiation and Functions of Osteoblasts by Runx2. Int. J. Mol. Sci..

[49] Elbjeirami W.M., Yonter E.O., Starcher B.C., West J.L. (2003). Enhancing mechanical properties of tissue-engineered constructs via lysyl oxidase crosslinking activity. J. BioMed. Mater. Res. A.

[50] Zhao Z., Qiao Q., Sun Y., Wang J., Li X., Zhang L., Yang H., Zhang K., Zhang N., Bai Y. (2025). Human periodontal ligament stem cells and metformin to enhance periodontal regeneration in rats. J. Dent..

[51] Yu Y., Mu J., Fan Z., Lei G., Yan M., Wang S., Tang C., Wang Z., Yu J., Zhang G. (2012). Insulin-like growth factor 1 enhances the proliferation and osteogenic differentiation of human periodontal ligament stem cells via ERK and JNK MAPK pathways. Histochem Cell Biol..

[52] Boda S.K., Almoshari Y., Wang H., Wang X., Reinhardt R.A., Duan B., Wang D., Xie J. (2019). Mineralized nanofiber segments coupled with calcium-binding BMP-2 peptides for alveolar bone regeneration. Acta Biomater..

[53] Yuen W.W., Du N.R., Chan C.H., Silva E.A., Mooney D.J. (2010). Mimicking nature by codelivery of stimulant and inhibitor to create temporally stable and spatially restricted angiogenic zones. Proc. Natl. Acad. Sci. USA.

[54] Browne S., Pandit A. (2014). Multi-modal delivery of therapeutics using biomaterial scaffolds. J. Mater. Chem. B.

