# Supplementary material for: Periodontal Regeneration Using a Mineralizing Hydrogel Loaded with a Synthetic CEMP1-Derived Peptide
Source: Biomolecules. 2026 Oct 3;16(10):1444. doi: 10.3390/biom16101444 (PMC13702467; doi:10.3390/biom16101444)
Supplement: Supplementary file 1 [file biomolecules-16-01444-s001.zip › biomolecules-4536395-supplementary.pdf]

Periodontal Regeneration Using a Mineralizing Hydrogel Loaded with a Synthetic CEMP1-Derived Peptide

Yu Wang [1], Guangzhao Guan [2], Li Mei [3], Huiwen Zheng [1], Xianghui Xing [1,*] and Weibin Sun [4,*]

[1] Department of Pediatric Dentistry, Nanjing Stomatological Hospital, Affiliated Hospital of Medical School, Institute of Stomatology, Nanjing University, Nanjing, 210008, China; 602023350107@smail.nju.edu.cn (Y.W.); zhangyipin@nju.edu.cn (H.Z.)
[2] Department of Oral Diagnostic and Surgical Sciences, Faculty of Dentistry, University of Otago, Dunedin, New Zealand; simon.guan@otago.ac.nz
[3] Department of Oral Sciences, Faculty of Dentistry, University of Otago, Dunedin, New Zealand; li.mei@otago.ac.nz
[4] Department of Periodontology, Nanjing Stomatological Hospital, Affiliated Hospital of Medical School, Institute of Stomatology, Nanjing University, Nanjing, China
* Correspondence: dr.xing@nju.edu.cn (X.X.); wbsun@nju.edu.cn (W.S.)

Supplementary Methods: qPCR analysis of hPDLCs treated with hydrogel extracts

Human periodontal ligament cells (hPDLCs) were seeded in 96-well plates and cultured overnight. The culture medium was then replaced with medium containing hydrogel extracts from the four hydrogel formulations (PEG Hy, ACP@PEG Hy, PM/ACP@PEG Hy, and P35/ACP@PEG Hy) for 7 days. The total RNA of the cells was extracted with RNA quick purification Kit followed by the synthesis of cDNA using the HiScript III 1st Strand cDNA Synthesis Kit (R312-01, Vazyme, China). The expression of osteogenesis- and cementogenesis -related genes (*RUNX2, OCN, CEMP1, and CAP*) was analyzed by iQ™ SYBR Green Supermix (Q511-02, Vazyme, China) with *GAPDH* as the internal control. The thermal cycling conditions were: initial denaturation at 95 °C for 30 s, followed by 40 cycles of 95 °C for 5 s and 60 °C for 30 s. Relative mRNA expression was calculated by 2^(-ΔΔCt) method.

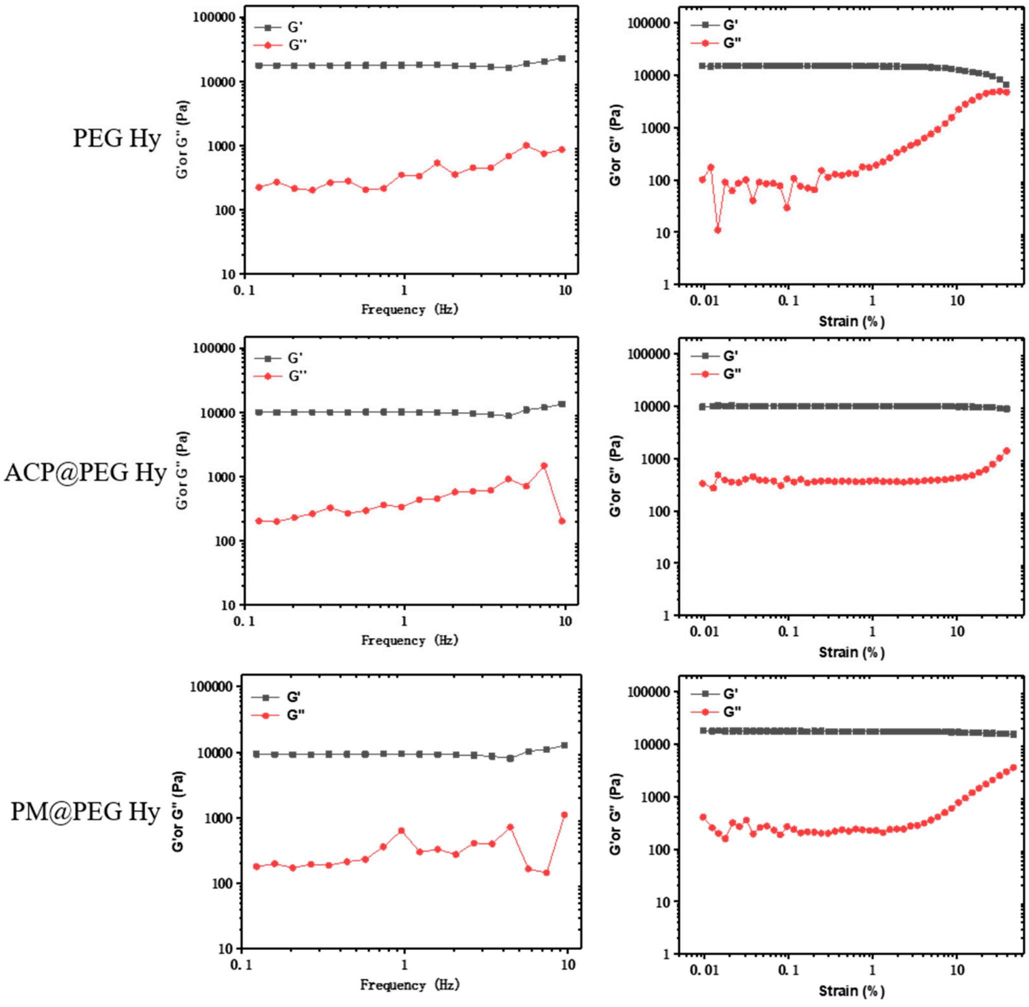

Figure S1. Frequency sweep test and Strain sweep test of PEG Hy, ACP@PEG Hy and PM@PEG Hy.

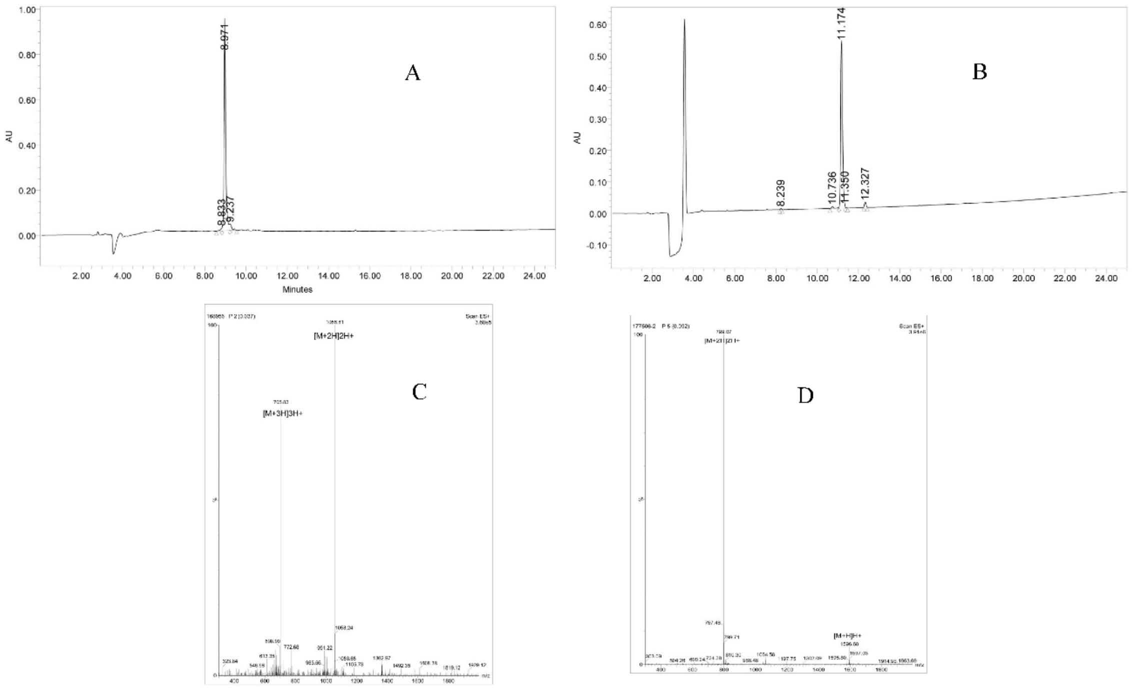

Figure S2. High-performance liquid chromatography (HPLC) and mass spectrometry (MS) analysis of the short peptide derived from the N-terminal 20 amino acids and C-terminal amino acids of CEMP1. (A) HPLC profile of the N-terminal peptide. (B) HPLC profile of the C-terminal peptide. (C) MS spectrum of the N-terminal peptide. (D) MS spectrum of the C-terminal peptide. The detected molecular weights are consistent with the theoretical values.

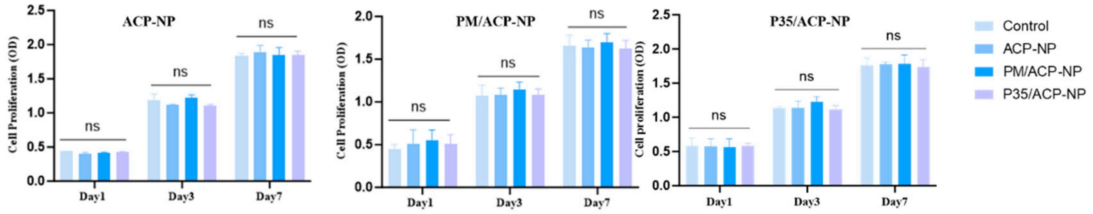

Figure S3. Cytocompatibility of nanoparticle formulations assessed by CCK-8 assay. Human periodontal ligament cells (hPDLCs) were treated with ACP-NP, PM/ACP-NP, and P35/ACP-NP at 10, 50 and 100 μg/mL for 1, 3 and 7 days. Data are presented as mean ± SD (n=3). Statistical significance was determined by One-way ANOVA with Tukey's post hoc test.

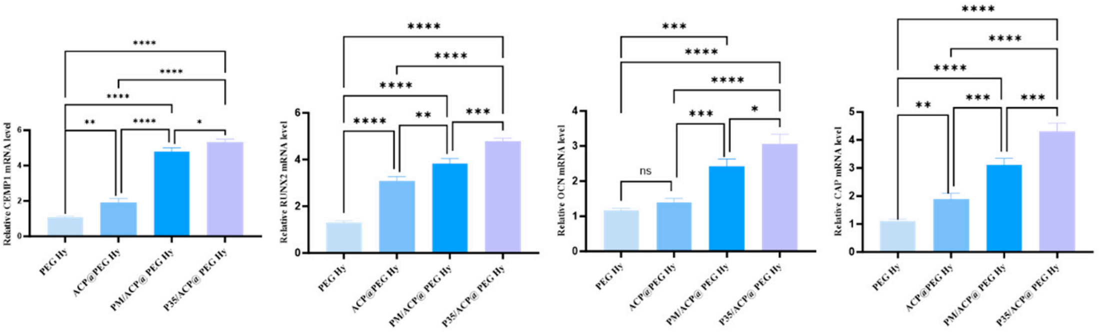

Figure S4. RT-PCR analysis of OCN, RUNX2, CEMP1 and CAP expression after 7 days of treatment with PEG Hy, ACP@PEG Hy, PM/ACP@PEG Hy, and P35/ACP@PEG Hy, respectively. P35/ACP@PEG Hy significantly upregulated the mRNA expression of osteogenic and cementogenic markers (RUNX2, COL1A1, OPN and OCN) compared to the other Hy formulations. Data are presented as mean ± SD (n=3). * p<0.05, ** p<0.01, *** p<0.001, **** *p<0.0001*.

Table S1. The primer sequence for RT–qPCR in PDLSCs treated with different nanoparticles.

Gene	Forward Primer sequence (5'-3')	Reverse Primer sequence (5'-3')
CEMP1	CCATCCTATCTCTTTGGACCTGG	CCTTGCTTACAGGTGCTGTCCT
CAP	CCTGGCTCACCTTCTCGAC	CCTCAAGCAAGGCAAATGTC
OCN	ATGAGAGCCCTCACACTCCTC	GCCGTAGAAGCGCCGATAGGC
RUNX2	CCCGTGGCCTTCAAGGT	CGTTACCCGCCATGACAGTA
GAPDH	GCATCACCGTCGAATCAAG	AAGCGCGATGGTGAAGACG

Table S2. Micro-CT parameters (mean ± SD, n = 8 per group) comparing P35/ACP@PEG Hy and PM/ACP@PEG Hy.

Group	n	BV/TV (Mean ± SD)	Tb.Th (Mean ± SD)	Tb.Sp (Mean ± SD)
Control	8	36.43 ± 2.31	0.115 ± 0.015	0.212 ± 0.010
PEG Hy	8	37.37 ± 2.35	0.118 ± 0.012	0.199 ± 0.004
ACP@PEG Hy	8	42.76 ± 2.23	0.141 ± 0.004	0.192 ± 0.005
PM/ACP@PEG Hy	8	50.38 ± 3.40	0.1542 ± 0.005	0.173 ± 0.003
P35/ACP@PEG Hy	8	56.72 ± 2.90	0.176 ± 0.004	0.160 ± 0.008
P value		< 0.001	< 0.001	< 0.001

P values determined by one-way ANOVA followed by Tukey's post hoc test. Overall $P < 0.001$ for all three parameters. Pairwise comparison between PM/ACP@PEG Hy and P35/ACP@PEG Hy: BV/TV $p = 0.0302$; Tb.Th $p = 0.0307$; Tb.Sp $p = 0.0431$.

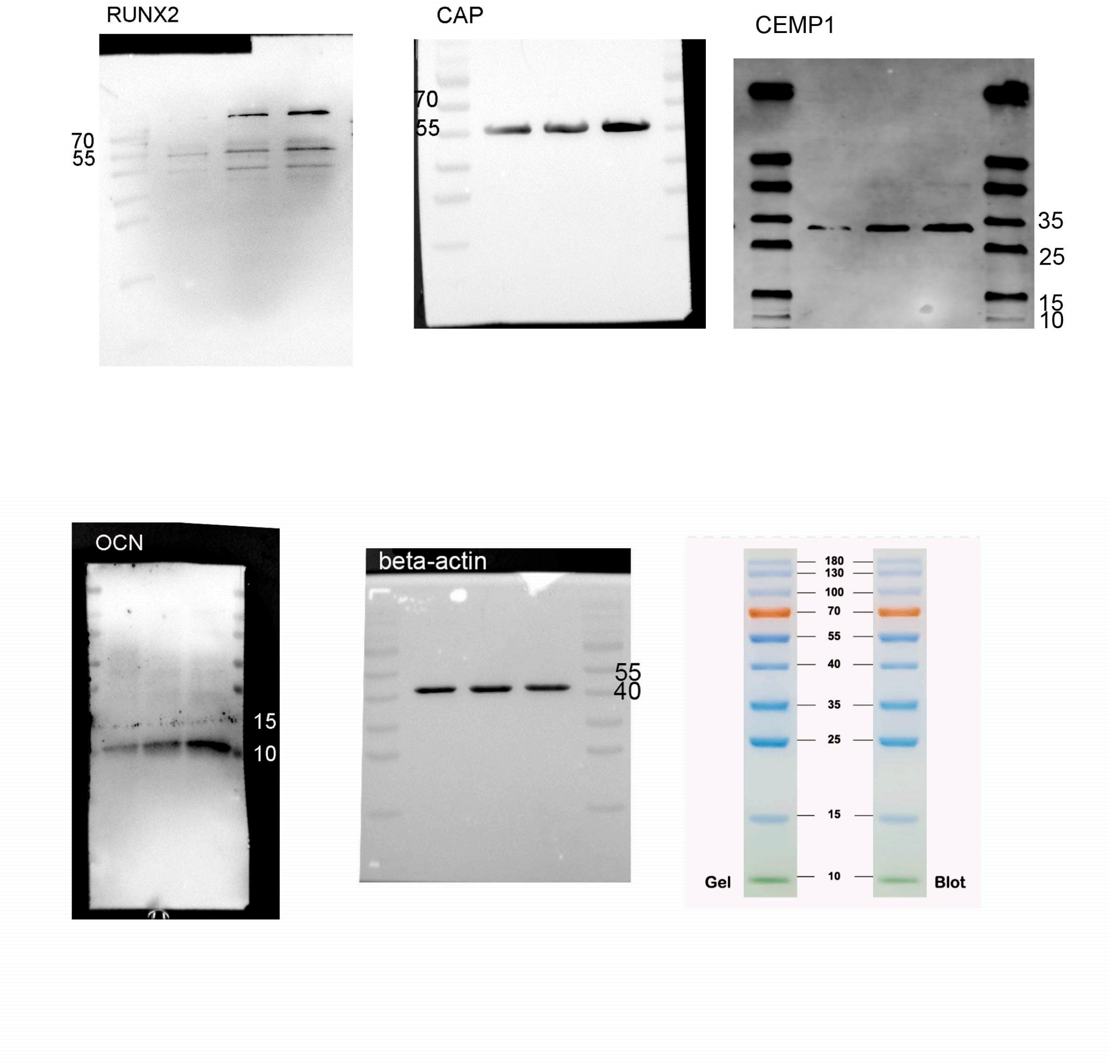

Figure S5. The original Western Blot membrane images of osteogenesis and cementogenesis related protein expression after incubation for 7days.

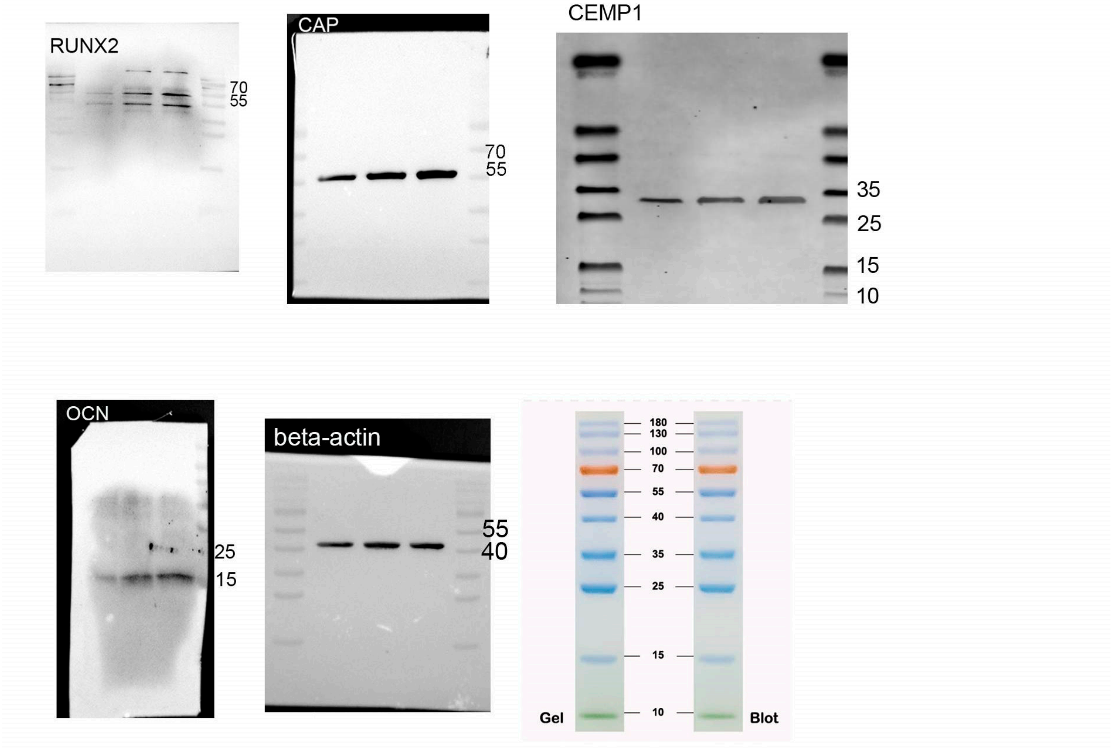

Figure S6. The original Western Blot membrane images of osteogenesis and cementogenesis related protein expression after incubation for 14 days.

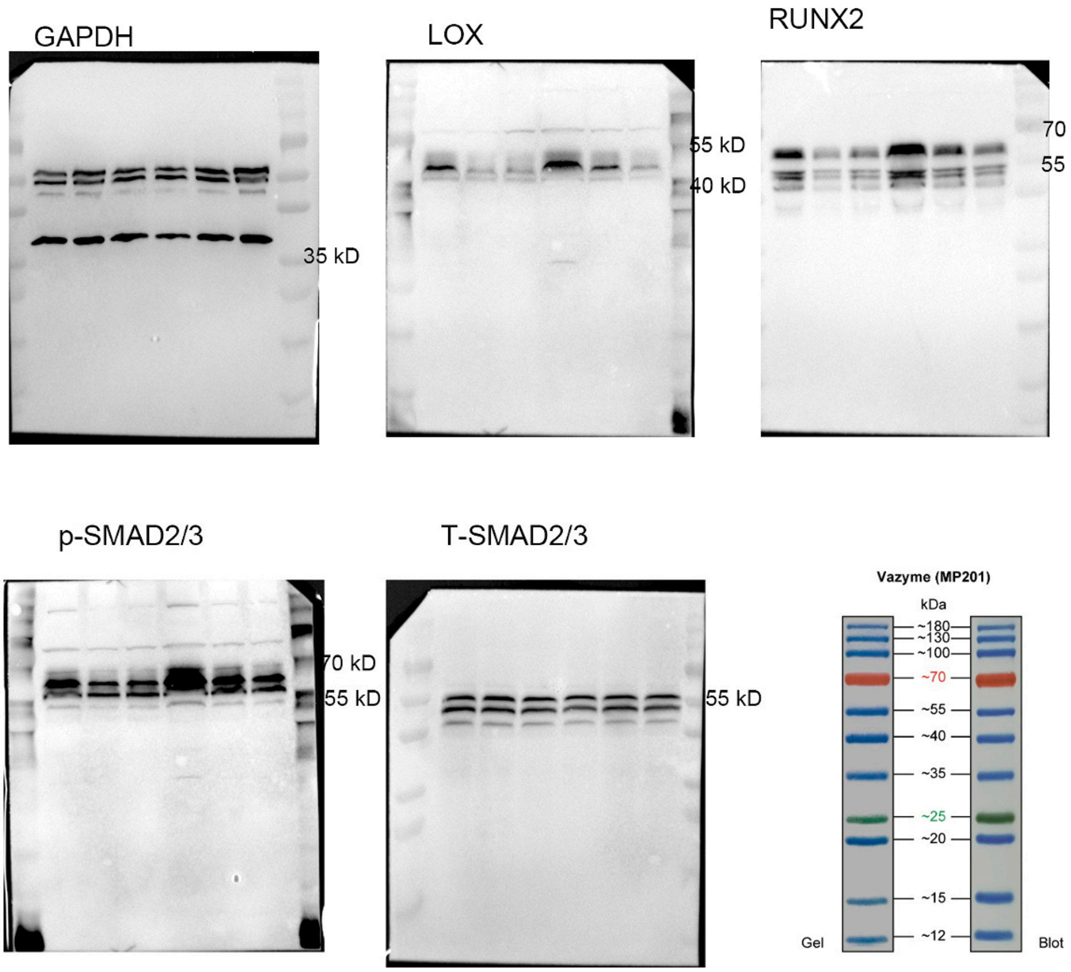

Figure S7. The original Western Blot membrane images of Figure 6.
